# Beyond retreat: Land–seascape legacies of change and continuation

**DOI:** 10.1007/s13280-025-02142-8

**Published:** 2025-03-25

**Authors:** Christina Hanna, Iain White, Raven Cretney, Pip Wallace

**Affiliations:** 1 Environmental Planning Programme, University of Waikato, Private Bag 3105, Hamilton, 3240 New Zealand; 2 Department of Environmental Management, Lincoln University, Ellesmere Road, Lincoln, Canterbury New Zealand

**Keywords:** Climate change adaptation, Disaster risk reduction, Land use change, Managed retreat, Post-retreat, Planned relocation

## Abstract

Planned retreat is an increasingly common climate adaptation approach. Whilst effective at reducing exposure to unacceptable risks, it is subject to significant resistance. In response, research has focussed on risk, governance and justice, but there has been less attention on spatial perspectives, including how spaces are reconfigured and the resultant land–seascape legacies. We identify 161 cases of planned retreat and develop a typology to analyse land use change in origin sites worldwide. In many cases, we find land transfer from private to public ownership, and restoration of land and environmental relations, to a complete failure to consider spatial legacies. The research stresses the importance of moving beyond planned retreat as risk management to consider longer-term spatial relations. Consequently, we argue, the definition and practice of ‘planned retreat’ must include early planning for the values, uses and reconfigurations of origin sites, extending the bounds of reimagination beyond ‘retreat’ and beyond the site.

**Supplementary Information:**

The online version contains supplementary material available at 10.1007/s13280-025-02142-8.

## Introduction

Among the various climate adaptation approaches, planned retreats will materially influence spatial functions and activities, thereby altering how communities use, perceive and relate to place. The range of retreats available reflects the diversity of environments within which they are applied. Determined by individual, community or governmental actors (Ajibade et al. 2022) and complicated by underlying histories, socio-economic contexts, culture and emotions (Moser 2021), planned retreats are a global phenomenon, occurring at various spatial and temporal scales and in differing ways.

From the advisory provision of risk maps that inform individual choices or insurance markets to more state-oriented policy, regulation or displacement, and financial incentives, researchers reveal how planned retreats can take place in multiple ways (Hanna et al. 2021; Ajibade et al. 2022). In some contexts, relocation or land use evolution may be a passive, intergenerational process; elsewhere, wholesale retreats have rapidly altered localities, such as via property acquisitions, buyouts, land swaps, abandonment, rezoning and rehabilitation of land (Hino et al. 2017; Lawrence et al. 2020; Mach and Siders 2021). Whilst strategies are initiated on a human-centred risk management basis and often define success relating to the removal of people and assets from unacceptable harm, retreats can also transition to projects that seek to restore ecosystems and enhance the liveability of communities.

The multi-faceted nature of planned retreats presents a formidable challenge for decision-makers. Often overwhelmed by the tasks and costs of managing risks and achieving relocation, the spatial functioning of origin sites (retreated areas) can be overlooked (Zavar and Hagelman 2016; Zavar et al. 2023). Yet, it is important to acknowledge that planned retreats have an ongoing legacy. Spatial reconfiguration or redesignation of what have been deemed ‘risky spaces’ (Haughton and White 2018) can have profound implications, from how nearby communities use that space, to what activities are allowed, to the potential to restore the health of ecosystems. Much research attention is focussed on risk management, policy and financial considerations of planned retreat (O’Donnell 2022). However, there is a parallel need to understand these initiatives as an attempt to remake space and place, that will have an ongoing social, cultural and environmental legacy that stretches beyond the site scale or a simple asset/risk perspective, to be more ‘relational’ (Hanna et al. 2022). A relational perspective turns the focus to how people perceive places. It emphasises that space and place are forever in production, with networks of social relations and meanings yet to be made, remade or not made at all (Massey 2006). Space and place are the ‘dimension of the social’, posing fundamental social, political, and ethical questions about how we coexist and how this changes over time (Massey 2006). The management of space and place, and ultimately how people live, identify and attach to place, entails responsibilities and consequences concerning how, where and whose relationships are fostered, protected or altered. This is the challenge of planned retreats and the remaking of place, and ultimately space. It is much more than the removal of people or assets from harm. How these risk management changes affect ongoing relationships to place can usefully draw from Massey's (1993) relational perspective, which shifts attention away from thinking of places as areas with boundaries, to allow for a sense of place that is multiple and extroverted, inclusive of its links with the wider world. Thus, any retreat also serves to alter networks of social relations and understandings (Massey 1993). This perspective provides a new dimension to retreat scholarship and allows for greater consideration of the spatial legacies risk managers leave behind.

The context and type of retreat will have differing spatial implications. Planned retreats often occur in the context of post-disaster activities and settings, including recovery, reconstruction and implementation of other mitigation measures (Zavar et al. 2023). Zavar et al. (2023) recognise that there are multiple experiences of retreat landscapes, from recovery to blight, which have impacts *beyond* the retreat zone (Binder et al. 2020). This is an important observation that highlights the value of a relational, spatial perspective. Planned retreats may create a transition in *landscapes* which can create a new sense of place and meaning, influenced by land use decisions, land management, and social engagement and interactions (Zavar et al. 2023). The concept of ‘landscape’, defined as ‘an area whose character is the result of the action and interaction of natural and/or human factors’ (ELC 2000) can provide an integrative spatial and socio-cultural platform (Galan et al. 2023) for planned retreat decision-making. By considering spatial–temporal dimensions including biophysical, social and cultural aspects, comprehensive planning can be promoted through the co-creation of landscape-based visions and relational spatial imaginaries (Walsh et al. 2022; Galan et al. 2023). From a planning perspective, this concept aligns with *relating* to the specific conditions of place, and to the promotion of more holistic and integrative planning (Wheeler 2002; Galan et al. 2023), making it suitable for addressing complex interrelations, and imagining adaptation (van Rooij et al. 2021).

There is growing awareness that adaptation planning cannot be realised without a coherent vision of the future landscape (van Rooij et al. 2021) or seascape (Gonçalves and Pinho 2024). Buyout-related terms such as ‘checkerboarding’ reveal both ad hoc retreats (as opposed to wholesale relocations) and the lack of a ‘landscape’ perspective, by ignoring the underlying socio-ecological systems. Aligned with relational concepts of the continual reproduction of ‘place’, Tsang and Stein (2021) argue that we must decouple concepts of land, loss and property value to recognise risky spaces as ‘lands-in-motion’, where there are opportunities among change. Importantly, planned retreats can support various societal goals, but decision-making and planning must operate at multiple, interconnected spatial scales to reflect biophysical and socio-cultural systems, involve multiple agencies and jurisdictions, address multiple risks and be strategically integrated into planning to achieve such goals (Carter et al. 2017; Siders et al. 2019). Whilst many forms of planned retreat to date may represent land loss or land use change, by developing a greater understanding of what comes next, it is argued planned retreats may be seen less as a ‘loss’ and more a significant opportunity to reimagine land use. Further, as global warming alters terrestrial boundaries, many low-lying coastal areas will experience transition to seascape (White et al. 2023). Analysing how retreats have led to diverse spatial outcomes and processes opens space to understand how they can support transformative social and political change, such as enhancing holistic well-being, breaking unsustainable land use patterns and transforming perceptions of climate change adaptation (Siders et al. 2021).This research is the first study to globally identify, categorise and analyse what happens to land and sea use post-retreat—were places lost or changed, or were land and seascapes completely reimagined? We identify and distil 161 international case studies to develop a typology that depicts the range of spatial functions, providing insights into optionality, and advancing understanding of what comes after planned retreat (Table S1). In doing so we suggest that narratives of retreat as ‘loss’ fail to capture the dynamic and complex outcomes that arise from these diverse forms of spatial reconfiguration. We emphasise the importance of moving beyond simplistic retreat or loss framings, which may exacerbate public resistance to change, to interrogate the theoretical, political, and social implications of planned retreats and encourage strategic adaptation. Central to our argument is the importance of questioning who benefits and what is altered, retained or even redressed through spatial reconfiguration, with a view towards building more just and equitable approaches to climate adaptation and land use change.

## Materials and methods

The development of a post-retreat typology involved an initial step of defining the scope of inquiry and the key characteristics. Planned or managed retreat is defined in this research as the relocation or removal of people, assets and activities to reduce exposure to climate and hazard related risks. We conducted a systematic literature review and content analysis to identify land use change types post-retreat following the PRISMA approach (Moher et al. 2009). The systematic bibliographic search included published, peer-reviewed articles on Scopus. A single database was used due to recent global systematic literature reviews undertaken on other databases and captured within the search results (Hino et al. 2017; Ajibade et al. 2022; O’Donnell 2022). As detailed in Table S2, the search terms were ‘managed retreat’ and ‘planned retreat’. Searches were conducted in March 2023, yielding 273 articles. Results were screened for data access and duplication: seven articles were not available online or via library sharing services and three duplicates were removed.

Results were filtered based on meeting the following criteria: planned/managed retreat had been implemented (but the post-retreat phase was either planned or implemented); the purpose included reducing natural hazard/climate risks to people or assets within national borders (thus excluding climate migration); case studies were present in the literature with sufficient detail on the location and approach. Due to the various ways in which planned retreats are termed and applied (e.g. managed realignment, relocation, land use change, resettlement), all 263 papers were read in full in search of reference to case studies. Ninety-eight records with empirical case studies were included in the dataset and coded by two coders. During the coding phase, references listed in papers were also reviewed where they were cited in relation to a case study. These were cited in Table S1 where they identified relevant post-retreat information regarding cases. Additionally, where data for a case was provided in partial detail during initial coding, supplementary internet searches were conducted to identify primary data, such as post-retreat master plans or project briefs, and cited in Table S1. In total, 208 academic and grey literature records informed the categories developed, with the total dataset producing 161 case studies.

Variables of interest (country, locality, number of households/hectares/infrastructure type, risk context and post-retreat information) were entered and data was manually coded alongside the descriptive data (see Table S1) to facilitate a systematic and rigorous approach. Functions of land/sea post-retreat were first coded using the wording in the article, then grouped with other similar codes and re-coded with a consistent typology (Table 1). In this second coding iteration, codes were developed to represent categories of spatial function in accordance with typical land use planning definitions and criteria (e.g. residential or conservation land). This allows a systematic comparison across national boundaries. For example, initial codes were changed from text such as ‘public reserve land’ to ‘Open Space’ in line with land use planning terminology. Each function was recorded once per case study, documenting the various types and presence of functions, not the spatial extent. Some case studies demonstrated multiple types and this was further noted as ‘multiple uses’ (with some exclusions, see Table 1). Commentary on the broad positive and adverse outcomes evident and not directly captured by the functional types was also coded (^). Following Ajibade et al (2022), coding involved a reiterative process, with information from additional sources enabling the description and code to be updated (and often validated) to reflect the latest published information. Coders also conducted a quality-control assessment to review and re-code any unreliable, incomplete or conflicting codes or data sources.

**Table 1:** Codebook with original and classified codes

Broad activity types	Specific coded category	Original case study activities/uses coded
Mixed use	*Open Space	Public river or coastal buffer easements, ecological parks/nature reserves, active and passive recreation areas, urban gardens, arboretum, walkways, parks, picnic areas, sports fields, skate and dog parks, fishing pier, canoe launch, and open-air pavilions, campgrounds, hiking and bike trails, baseball park, accessible recreation improvements, community gardens, orchards and foraging zones, maritime forests, wildlife observation areas, pollinator parks, contemplation sites, boating, museum, lake, educational facility, riverside park (with picnic areas, tennis courts and campgrounds), parking lot for park, urban wildlife habitat enclaves, cross-country trails, exhibition centre and kiosk, park leases for social enterprises, wheelchair and family accessible nature reserves, reserve land cafes and visitor centres, cultural gardens, public art and environmental education spaces, and more limited transitory recreational parks (due to risk to human life), flood water retention and recreation spaces, unmaintained open spaces, greenway with golf course, fishing sites, trails, boat ramps and campground, park with community assets, basic open space, environmental or community based open space, open space farming leases or agricultural based land management, mowed grass and planted open space, beach access way, estuary, open space zoning allowing for: fungi farm, river sports, eco-hub, playground, berry farms, propagating apricot trees, cropping, grazing, keeping beehives and chickens, and nursing plants, nature zones, memorial garden, BMX track, dog parks, coastal park, social enterprise
	*Redevelopment	Housing upgrades, neighbourhood revitalisation, elevated housing, residential housing, adaptable housing, social housing, infrastructure upgrade, urban esplanades, adaptive urban infill, new urban amenities, roading, sewerage, pedestrian and cycling bridges, affordable housing redevelopment, stream canals and dams, housing development following flood pump and drainage improvements, private development *Note: We recognise that the category ‘redevelopment’ may fit more broadly under adaptation than retreat, but in the cases identified there has been removal of the original activity or structure, with predominant replacement with a new form of activity*
Conservation	*Floodplain restoration	Floodplain restoration, reconnection, protection and associated habitat restoration, river restoration planting
	*Coastal restoration	Saltmarsh restoration, reconnection, protection and associated habitat restoration, sand dune restoration and planting, mangrove planting, greenbelt planting, regenerating coastal forests and igune, restoring living shorelines, restoring maritime forests and tidal wetlands, beach nourishment, marine protected area
	*Ecological restoration	Ecological park rehabilitation and restoration planting, creation of urban wildlife enclaves, grazing ban to restore the ecological environment, riparian and native wildflower planting
Risk reduction	*Blue–green infrastructure	Floodwater retention and detention areas, purposeful natural buffers (*living shorelines, vegetative flood buffer, willow forest, maritime forests, coastal greenbelts, igune, greenway, sea grass, increased land surface roughness to reduce wave runup energy, dune systems, saltmarsh restoration, coastal lagoon and wetlands*) overflow paths, sand replenishment, erosion and sedimentation planting, managed realignment, water retention tree planting, riparian planting, river flow restoration, esplanade and drainage reserves, wetlands
	*Protection measures *Protection and accommodation measures	Levees, removable floodwalls, dikes, tide gates, breakwater reefs, seawalls, pumping stations, embankments (protection) Elevated roads, evacuation hills and towers (accommodation)
Rural	*Farming continues	Land has remained in agricultural use
Heritage	*Heritage preservation	Preservation of heritage sites and/or cultural landscapes (see Table 4)
Residential	*People remain in situ	People remain in the retreat zone due to voluntary and involuntary immobility, incomplete/staged buyout programmes, lack of business relocation support, place attachment and cultural relations
	*Residential reoccupation	Reoccupation of retreat zones due to limited enforcement of regulations
	*Residential leasebacks	Leasebacks for older residents or people who need more time to plan relocation and orphan parcel leases for nearby property owners
	*Residential amalgamations	Property sold to owners with neighbouring lots
	*Residential setbacks	Privately owned structures relocated within a site
Commercial	*Commercial	Golf course, retail, home business, car parking, motor caravan park, campgrounds, community and business leases
Vacant land	*Abandonment	Abandoned villages, schools and ‘ghost towns’
	*Demolition	Demolition of buildings leaving vacant land and phased, generational demolition of buildings
Sea change	*Sea change—Transition brought about by the sea	Abandonment of defences and agricultural land to mangroves, land lost to the sea, erosion continuing to encroach on village, land is wet at full tide
**Secondary coding layers:**	Multiple uses	Two or more purposeful functions that are not present by default (e.g. this excludes *people remain in situ where it is an unplanned function and *coastal restoration of dune and public beach *open space multi-functions)
	Multiple uses—networked	Strategic, integrated landscape planning
	Outcomes ^	Broad positive and adverse outcomes evident in the literature that are not directly captured by the functional uses

Overall, 19 post-retreat categories were identified across nine broad activity types. The validation and refinement of this data led to the inductive development (Thomas 2006) of the four broad themes designed to better communicate patterns of post-retreat (Table 2), and a conceptual framework to broadly examine the nature of change (Fig. 2). These concepts are heuristic, enabling the comparison of the large number of case studies and the diverse outcomes documented. To examine the spatial legacies of post-retreat environments, the conceptual model broadly outlines the spectrum of ‘lands in motion’ (Tsang and Stein 2021) that was evident across the case studies, from ‘land loss’, and ‘land use change’, to ‘land/seascape imagination’. There were clear cases of land loss which can be situated to the left of the spectrum, such as the ‘abandonment’ and ‘sea change’ types identified in the fourth theme (see “Results”). But for other cases, the difference between land use change and landscape reimagination required further analysis.

**Table 2:** Dominant legacies and outcomes of origin site functions

Broad retreat legacies	Documented outcomes of origin sites
Nature-based solutions (defined as approaches to restore, protect, reconnect*,* or manage natural and modified ecosystems to address societal challenges)	*Ecosystem functioning:* Floodplain and saltmarsh restoration, reconnection, and protection, dune restoration, groundwater recharging, sea grass acreage increase, sediment storage capacity increase, rehabilitation, river restoration, water quality improvement, removal of hazardous, deteriorating coastal protection measures *Ecosystem adaptation:* Ecosystem migration space afforded, compensatory habitat provided for coastal squeeze, species relocation, abandonment of protection measures allowing natural mangrove migration, strategic conservation to support the transition of uplands to marsh *Biodiversity:* New, restored, enhanced, reconnected and protected habitats, and improved biodiversity outcomes *Blue–green infrastructure*: Floodwater retention and detention areas, natural buffers (living shorelines, vegetative flood buffer, willow forest, maritime forests, coastal greenbelts, igune, greenway, sea grass, increased land surface roughness to reduce wave runup energy, dune systems, saltmarsh restoration, coastal lagoon and wetlands) overflow paths, sand replenishment, erosion and sedimentation planting, managed realignment, water retention tree planting, riparian planting, river flow restoration *Climate change mitigation potential:* carbon accumulation
*Preservation* of cultural land and seascapes, heritage sites, livelihoods*,* and land uses (defined as selected preservation and protection of cultural values, livelihoods and land–seascapes, and activities and infrastructure)	*Cultural landscapes and heritage site preservation/repurposing:* Ensuring continued access to ancestral lands/villages/livelihoods*,* heritage site protection providing access to old farm lands and grave lands; historic buildings prioritised for relocation to help preserve cultural heritage; heritage protection in situ; coastal heritage project to help the local community know and record the threatened heritage so they can interpret the village’s historic, cultural and environmental landscape; heritage building repurposing; cultural trails; Indigenous vegetation planting to revive cultural food practices and landscapes; applying cultural and historic design principles; pow-wow grounds and Church in origin site; mahinga kai (Indigenous Māori food-gathering environments), cultural trails, heritage house and gardens; archaeological excavations under licence; memorials and plaques—disaster and relocation history; pā harakeke (Indigenous Māori weaving and flax plantations), te māra rongoā (Indigenous Māori medicinal gardens), pou ihi (Māori carving), environmental education—Māori worldview *Protection-based infrastructure:* Evacuation hills and towers, levees, removable floodwalls, dikes, tide gates, breakwater reefs, seawalls, pumping stations, embankments, elevated roads *Land use continuation*: people remaining in situ and leasebacks
*Socio-economic opportunity* (defined as post-retreat functions that are focussed on enhancing social and/or economic outcomes)	*Community enhancements/amenities:* Active and passive recreation, urban gardens, arboretum, walkways, parks, picnic areas, sports fields, skate and dog parks, fishing pier, canoe launch, and open-air pavilions, campgrounds, hiking and bike trail, accessible recreation improvements, community gardens, orchards and foraging zones, maritime forests, wildlife observation areas, beach restoration, pollinator parks, contemplation sites, boating, museum, educational facility, riverside park, increased yard space, nature reserves, parking lot, tree planting, urban wildlife habitat enclaves, cross-country trails, exhibition centre and kiosk, leases for social enterprises, nature information stands, wheelchair and family accessible nature reserves, cafes, visitor centres, cultural gardens, art and environmental education, and more limited transitory recreational parks (due to risk to human life) *Food provision:* Agriculture, horticulture (corn farming, community gardens and orchards, fungi and berry farming) apiculture, chicken keeping, plant nurseries and propagation (apricot trees), foraging zones and agricultural land rehabilitation post-disaster (remove rubble and salt, restore drainage and pumps), fishing, mahinga kai restoration and te māra rongoā *Urban development and demolition* (reduction in potential for or presence of blight): housing upgrades, neighbourhood revitalisation, elevated housing, residential housing, adaptable housing, social housing, infrastructure upgrade, urban esplanades, adaptive urban infill, new urban amenities, roading, sewerage, pedestrian and cycling bridges, affordable housing redevelopment, stream canals and dams, housing development following flood pump and drainage improvements) *Tourism*: Environmental, adaptation, and heritage-based tourism (nature reserves, environmental education facilities, museum, cafes, retail, temporary land leases), beach restoration supporting tourism *Offsets:* Managed realignment restoration activities used to offset unavoidable development or infrastructure-related losses in a region *Economic:* reduced risk management and structural protection maintenance costs, urban development, avoided flood losses, job creation, resource repurposing, commercial activities (golf course, retail, home business, car parking, motor caravan park, campgrounds, community and business leases), economic benefits from horticultural and agricultural leases, yard maintenance leasebacks reducing open space management costs, greenway acting as an anchor for investment/revitalisation in the city and redevelopment *Human health and safety benefits:* Water infrastructure improvements and improved health conditions by avoiding frequent floods with raw sewage and mosquito transmitted diseases, removal of hazardous, deteriorating coastal protection measures and land rehabilitation *Education and research* (adaptable housing, climate change adaptation, blue carbon), fungi farm education, eco-hub, pā harakeke and pou ihi, conservation education
*Vacancy, abandonment*, *and harm* (defined as limited, inequitable and/or maladaptive post-retreat planning)	Vacant, unmaintained or abandoned sites contributing to blight, ‘Sea Change’ abandonment, environmental and safety concerns; people remaining in situ and at risk or with limited services and support; reoccupation of hazardous areas; redevelopment enabling disaster capitalism and displacement; ‘green grabbing’ and disruption to food production and livelihoods and reduced access to Indigenous food practices; environmental destruction (mangroves and sedimentation) and sewerage and landfill erosion in origin sites; inequitable investments and justice concerns regarding land use change; reduced access to open space lands during/following managed realignments; impacts on peripheral communities

At this point, case study documents were re-reviewed to examine the approach to planning the post-retreat environment with a particular search for strategic, integrated, multi-scalar planning to support reimagination of land and seascapes. Two key questions underpinned this qualitative analysis: Does the case study demonstrate features of an integrated approach to achieve multiple outcomes, and are the retreat outcomes multi-scalar or landscape/seascape based, reaching beyond the subject site/s (Carter et al. 2017; Siders et al. 2019; Walsh et al. 2022; Galan et al. 2023)? An additional code subset ‘networked’ was applied to cases demonstrating a strategic and multi-scalar approach to post-retreat planning, integrating various ecological, socio-cultural, and political outcomes, and this was also detailed in the case description column (see Table S1).

Limitations of the research include the use of secondary data for the case studies, which has been supplemented with primary data where available. We recognise that due to the discrepancy in data types, there may be other functions and planning features than what we have captured, highlighting that this research provides a representation of what has been recorded primarily in the academic literature, and that there can be limited data on these elements. Furthermore, as evidenced by the large number of English language dominant case studies, and in particular, case studies from the USA and England, the results are more relevant to these nations and highlight the value of multi-lingual research to improve representation and diversity of results. In particular, the managed realignment case studies are generally cited from physical scientific literature, resulting in limited information about open space/public land uses, hence the use of public reserve information. We also recognise that there are limitations in relying on literature to identify land uses which may evolve over time, and therefore this research presents a recorded snapshot, not necessarily the final state of retreat land/seascapes. Finally, authors of the studies may influence what is being recorded in the case studies. To minimise potential biases in reporting and increase reliability, where available, we examined multiple academic and planning materials for each case study, cross-referencing and updating our findings where appropriate. Whilst the list of case studies is significant, it is not exhaustive, and therefore we do not consider these functions to be exclusive and encourage others to further develop this work.

## Results

### Legacies of change and continuation in origin sites

**Fig. 1:**
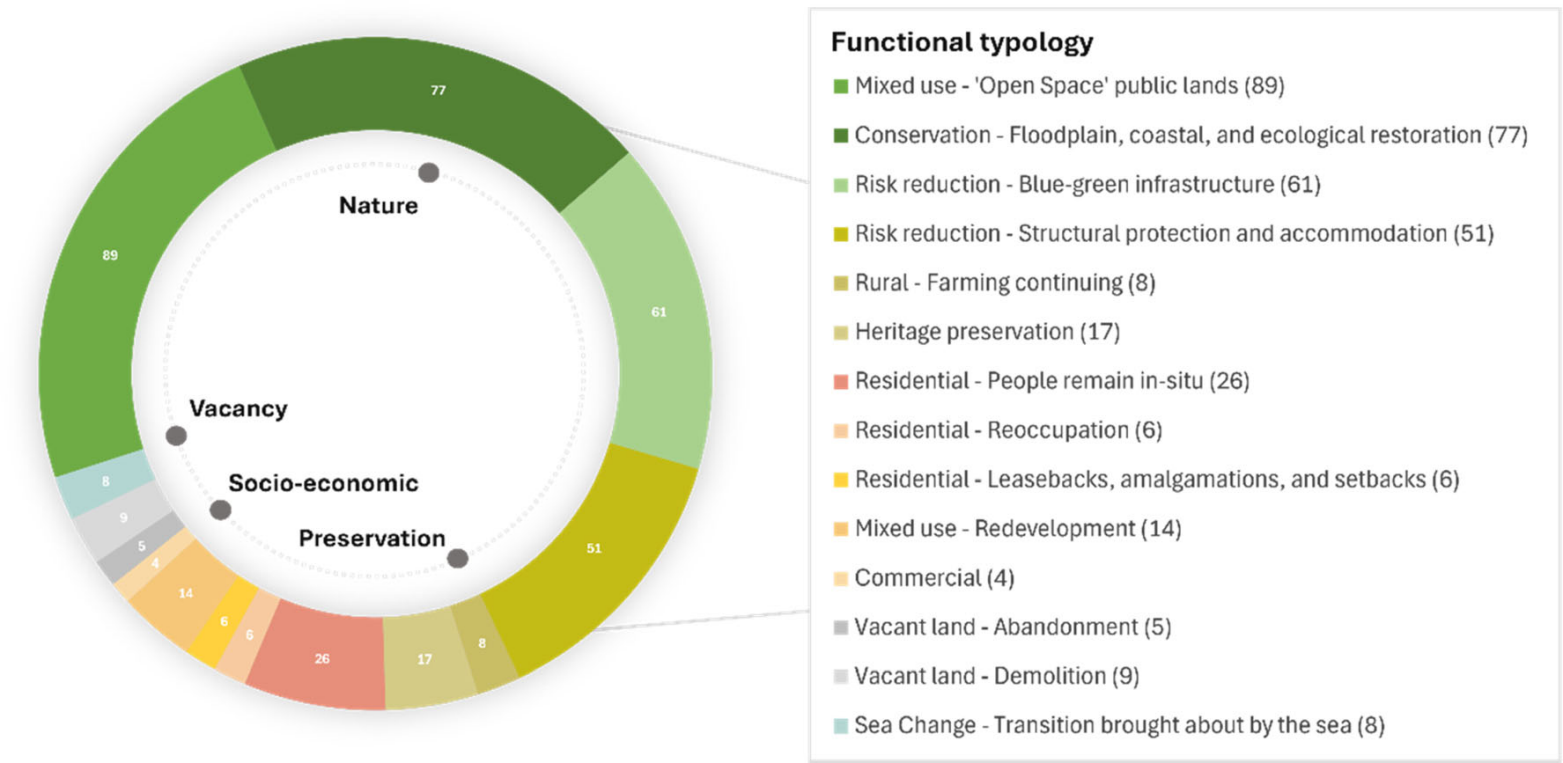
Functional typology of global origin sites (in colour)

Figure 1 presents the organisation of planned and implemented case studies into categories of post-retreat spatial functionality, in accordance with typical land use planning criteria. Data is based on functional presence, not spatial extent.

The typology categorises diverse approaches and provides new insights into the patterns of land/sea use post-retreat. A total of 161 global case studies provide evidence of the differing spatial reconfigurations produced, with a transition from residential, community, farming, and business properties to conservation, open space and vacant lands, or sea. In some cases, redevelopment, reoccupation, and leasebacks have enabled continued residential or mixed-use activities, and farming has remained. Heritage preservation and risk reduction functions highlight the multiple uses of origin sites, often complementing conservation and open spaces.

The typology allows us to identify four broad retreat legacies: nature-based solutions; preservation of cultural land and seascapes; socio-economic opportunity; and vacancy, abandonment, and harm. These themes are not exclusive, and can overlap, such as ‘nature’ and ‘preservation’, with heritage preservation occurring in nature reserves. These retreat legacies synthesise functions into new categories that demonstrate how global practitioners and communities have sought similar outcomes (Table 2), communicating the dominant spatial patterns post-retreat.

### Nature-based solutions

The data reveals that rehabilitation of origin sites commonly embraces nature-based solutions. Case studies demonstrate significant conversions of private to public land, with conservation (*n* = 77), mixed-use open space (*n* = 89) and blue–green infrastructure (*n* = 61) functions representing the primary uses of post-retreat environments. Ecosystem restoration is common, with many sites rehabilitated, and floodplains, rivers, and coastal ecosystems restored, revegetated, and/or reconnected. This conservation-based category often delivers allied functions, such as recreational nature reserves (e.g. see Abbotts Hall, England, #109, Table S1). Mixed-use open space lands are public lands with a range of potential uses such as nature, community, storm water, or recreational reserves, and farming, often with regulations restricting structural development or risk sensitive activities (see Matatā, Aotearoa (New Zealand), #158, Table S1). Planning and investment in these areas range from basic land rehabilitation (e.g. grassed sites) to established parks and reserves, such as the Grand Forks (USA) riverfront greenway which hosts various recreational courses, connected trails and flood protection measures, alongside riparian planting and wildflower restoration (#54, Table S1).

Nature-based solutions can provide an ongoing risk reduction legacy beyond the primary task of reducing exposure to harm. Blue–green infrastructure (see codes in Table 2, for examples) is a key function of the sites studied, indicating the combined utility of reducing direct human/asset risk exposure whilst achieving risk reduction beyond the origin site/s. For example, the introduction of living shorelines (Punta Gorda #50), willow forests (De Noordward #102), and coastal igune (Japan #20) to buffer storms, or flood water retention (Iowa, #43) in open spaces, compounding adaptation benefits. With thousands of hectares of wetland restored, planned retreats may also support climate change mitigation, but the origin, nature and long-term accumulation of carbon is pertinent (Mossman et al. 2022).

Improved ecosystem outcomes are therefore an important retreat legacy identified. Globally, many case studies aim to restore ecosystem functions, support biodiversity and in a few exceptional cases (n = 6), create space for ecosystems to adapt and migrate (e.g. space for ‘marsh migration’ Mastic Beach, USA and ‘habitat transition’, Medmerry, England. See cases #48, 60, 79, 113, 122 and 137 in Table S1). These examples stretch the purview of retreat beyond anthropocentric functions, realising both intrinsic and instrumental values of ecosystem adaptation and nature-based solutions. However, we recognise that conservation efforts may not always equate to biodiversity gains, due to the value of these sites as ‘offsets’, such as for infrastructure projects, and offset policy design. The use of offsets emphasises the economic potential of planned retreats, but dynamic environments require careful policy attention, with "inherent challenges and uncertainties in recreating new habitat of the same quality and composition as that which is lost” (Brown 2022). Retreat analysis predominantly focuses on human impacts, yet it alters the habitats of other species too, such as the relocation of badgers and snakes (May and Smart 2003). Advancing the ecosystem legacies of planned retreat requires “long-term strategies and interventions on a landscape scale to emphasise ecological functioning, integrity, and coherence, consistent with an ecosystem-based approach” (Brown 2022).

### Preservation legacies

Continued agriculture (*n* = 8), heritage preservations (*n* = 17), protection and accommodation measures (*n* = 51), and social–cultural activities and connections (see cases in Mozambique, Fiji, and the Solomon Islands, for example) in origin sites demonstrate that planned retreats are not always full and final withdrawals from place. Instead, they represent an adapted relationship, with sensitive activities relocated and other practices remaining, preserving cultural values and spatial functionality. An important and potentially overlooked function of origin sites is continued human access, particularly for Indigenous communities, to preserve environmental relations and cultural legacies. Whilst not widespread practice, it is evident that protection of origin site access is becoming recognised as an important retreat principle (see Fijian cases 144 and 146 in Table S1 and Ministry of Economy Republic of Fiji (2018)) to maintain place attachment and protect environmental relations.

Demonstrating temporary preservation of land use, two case studies (North Carolina, USA and Waitakere, Aotearoa NZ) applied residential leasebacks for property owners, allowing them to remain in situ for a set period of time. These highlight the transitory use of land where retreat is necessary to cease private property rights and investment in risky localities, but existing relations are accommodated in the near-term, to avoid detachment from place for older residents. In North Carolina, orphan parcel leases were also provided to neighbours for use of vacant sites indicating the potential for sharing the maintenance burden and supporting connection to place among those who wish to utilise these spaces. Residential amalgamations (*n* = 2) were available in two buyout programmes in New Orleans, USA, with some property owners able to purchase adjacent vacant lots (see cases 58–59 in Table S1). Preserving the status quo in some sites, partial relocations (*n* = 26), building setbacks (*n* = 2) and reoccupations (*n* = 6) highlight the importance of considering people who remain in situ, amidst a changing or abandoned landscape, lacking services, security or support.

### Socio-economic opportunity

In contrast to preservations, planned retreats can provide opportunity to remake places, with risk reduction measures addressing legacy issues and enabling future redevelopment. Here, the land use function may be unchanged but opportunities to reimagine the urban landscape arise. Urban development (*n* = 14) in origin sites has built new urban waterfronts and esplanades, improved infrastructure and enabled comprehensive planning for new educational, cultural and tourism amenities. In addition to reduced risk management costs, tourism and development outcomes highlight the economic opportunities of planned retreat. In one case, the transformed landscape features as the tourism attraction (Biesbosch Museum, De Noordward). Sites have also been commercially developed (*n* = 4) for golf (Indiana, USA) and agribusiness (San Martin, Peru), for example.

In the open space zones, community amenities, such as cycle trails, educational fungi farms and accessibility friendly parks have been developed, or campgrounds are present, emphasising that risk management continues post-retreat. The case studies also highlight that origin sites can increase opportunities for local food networks (see ‘nourishing lots’ and ‘edible foraging’ zones in Aotearoa and Canada cases #35,149, 156 and corn farming in the USA, #41). Ex-residential sites, with mature fruit trees and residential gardens, can bestow a foundation for community orchards and food foraging zones. Whether retreat sites have a significant food legacy will depend on the ability/need to remediate land and the regulatory support for food production, particularly in open space zones. However, with land use change, or in a post-disaster or climate changed setting, site rehabilitation and remediation may be required. This is a challenge for origin site use generally, particularly if there is considerable development, legacy landfills or contaminated soils to remediate.

### Vacancy, abandonment and harm

Where there was no post-retreat planning or site rehabilitation, areas have been abandoned (*n* = 5), leaving eerie reminders of the costs of living in danger zones (i.e. the ‘ghost town’ Shawneetown, Illinois #63). This may be due to a failure of support, budgets or political imagination, viewing success from a narrow risk management perspective, or a reliance on private insurance rather than state-led action, as many spaces hold potential to be reimagined. In some contexts, these reminders are purposeful monuments reinforcing the permanence of retreat. Elsewhere, demolition of structures was indicated (*n* = 9), but no further action evident, indicating vacant land. Eight case studies demonstrate a ‘Sea Change’ with land becoming foreshore and sea. In Denimanu, Fiji, the eroding shoreline continues to move into the village despite previous relocation efforts, with houses flooding during spring high tides (Martin et al. 2018). In Newtok, Alaska, land is steadily disappearing due to increasing temperatures, coastal erosion, and thawing permafrost. As residents transition away from Newtok, preserving their cultural heritage and traditions is a significant concern, as is the protection of human rights (Bronen 2014). Emphasising further risks of retreat, some redevelopment examples exhibit displacement under the guise of planned retreat (Chennai, India #7), and disaster capitalism, with selective retreat enforcement for fishing communities, but not commercial/tourism activities (Sri Lanka, #18). In Zambia, the Namapande Resettlement Scheme and ‘adaptation agenda’ is considered a means for the state to legitimise control over customary lands and production (Funder et al. 2018). In terms of the spatial legacies to avoid, case studies identified a range of adverse outcomes including risks to people who remain in place, due to service withdrawal (Mozambique #2), lack of consideration of peripheral communities (e.g. Staten Island, USA #62), disruption to food production and livelihoods (e.g. Chennai, India, #7) disaster capitalism (Sri Lanka, #18), environmental destruction from relocation activities (Narikoso, Fiji #145) and equity concerns regarding investment in and maintenance of origin sites (Harris County, USA #83). These outcomes highlight the importance of attention to intersectional justice and equity dimensions (Ajibade et al. 2022) from relocation to repurposing, and to consider boundary effects on ‘safe’ neighbouring communities, to avoid legacies of societal and environmental harm.

### Regional trends

Table 3 summarises the regional trends according to presence of functions and risk drivers of retreats (see Table S3 for national breakdowns). The regional distribution of functions aligns with the overarching trend of conservation and open space land use for origin sites. Whilst there are notable differences, such as ‘reoccupation’ of retreated areas being observed almost exclusively in Asia (with one North American case study in Leavenworth, Illinois—1938), we acknowledge that the data will not fully represent all retreats across different nations or regions (refer to Methods). However, an examination of functional presence reveals clear similarities among Asia, Europe, North America and Oceania regarding the diversity of land use functions applied. Regional comparisons also highlight the varying contexts of planned retreats worldwide. For instance, in Europe, coastal managed realignment projects create distinct retreat environments with a strong emphasis on conservation and environmental offsetting opportunities. Conversely, disaster event response and housing-related retreats in the USA, Aotearoa and Australia tend to apply mixed-use open space zones, likely due to the residential contexts in which retreats have been focussed (see Table S1). With emerging examples of supported ecosystem migration, these patterns may evolve over time, ideally leading nations to demonstrate varied forms of planned retreats and origin site use to address holistic well-being. In terms of risk drivers for retreat, coastal and riverine risks are the most prevalent, with some instances of hurricane and geological risk responses. Notably, the regional data indicates that countries with a higher number of case studies exhibit a greater diversity of land uses, emphasising the importance of further primary research. Finally, care should be taken in reviewing Table 3 as whilst there is ‘presence’ of certain functions such as heritage preservation, this remains a limited feature of the case studies and deserves much greater attention in future.

**Table 3:**
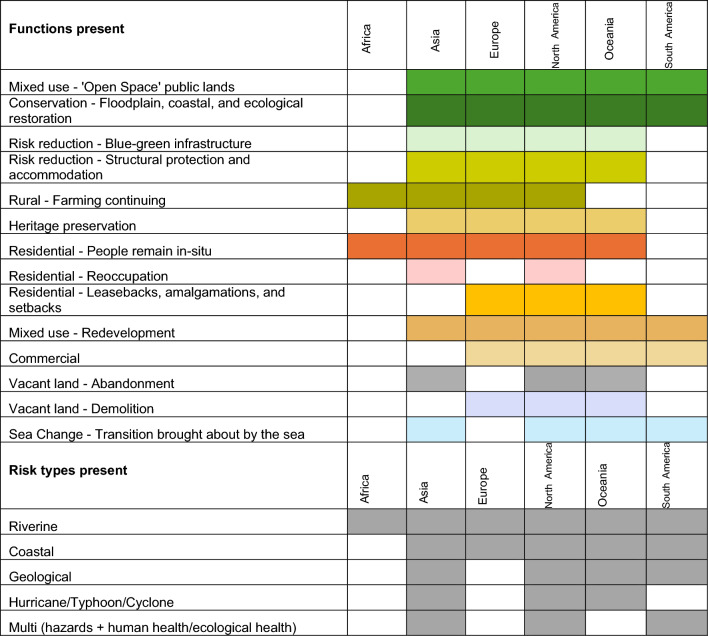
Regional trends—functions and risk types

## Discussion

**Fig. 2:**
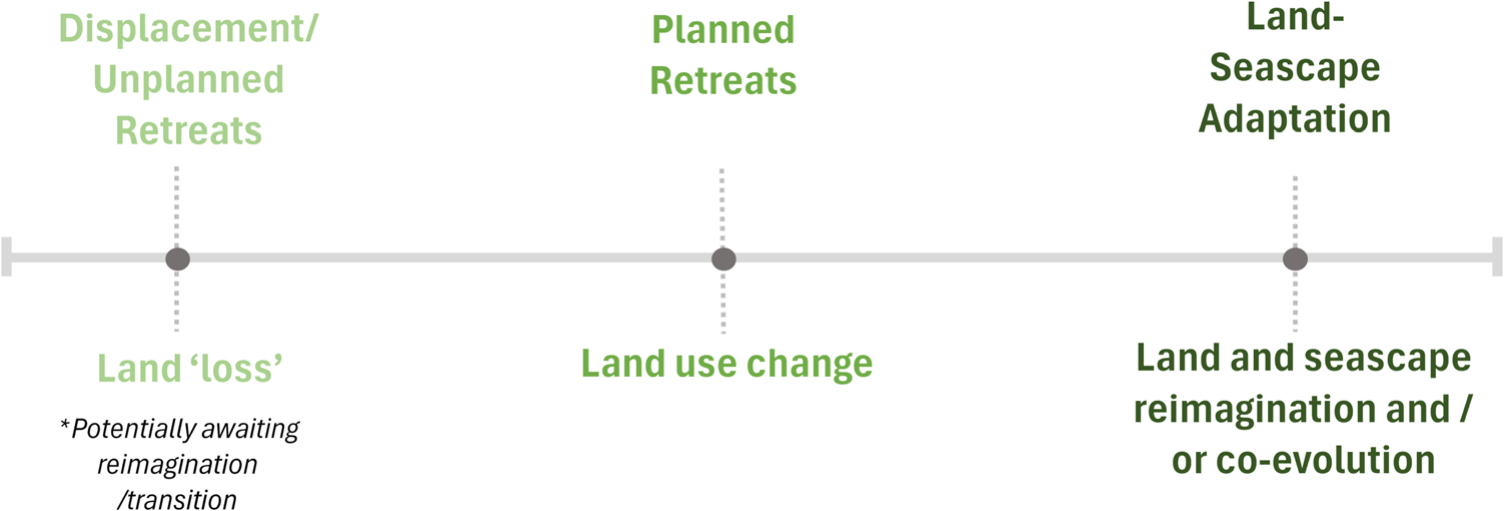
Loss to reimagination: Spatial legacies of retreat

The “Results” section identified types and themes of post-retreat activities, but there is more to this story than the functional categories of land and sea use. As an opportunity to remake space, retreats have implications for how we perceive, value and use places. To conceptualise the broad *spatial* influence of origin sites and their functions, Fig. 2 depicts the scope of *spatial change* identified in the case studies, from ‘land loss’ and ‘land use change’ towards ‘land and seascape reimagination/co-evolution’. ‘Land loss’ occurs under ‘Sea Change’ without plans to manage and support the transition and relations. Where partial relocation occurs, people remaining in situ without services and support experience reduced capacity to remain, which may amount to land utility loss. Here, post-retreat environments are less able to attend to societal goals and relations. We recognise, however, that there are temporal, political and cultural elements at play, and experiences of ‘lost’ and ‘vacant’ areas may evolve over time, holding their place as ripening fruit for future repurposing, or as passively restored wild spaces resulting in gradual biodiversity gains (Daskalova and Kamp 2023). Perceptions of loss will depend on worldviews; some may not consider ‘sea change’ a ‘loss’, or this may depend on the protection of environmental relations and governance. Finally, we recognise that some lands will not have a safe use post-retreat. Human land utility/access loss is appropriate in some places, and not all land needs to be designated for, or managed by humans.

The case studies analysed largely demonstrate the middle ground, ‘land use change’, where land use was intentionally changed, post-retreat. Here, planning is often constrained to the site scale, with isolated functions such as basic naturalisation of open space areas or vacant lots. Whilst the specific utility or maintenance of all sites was not always clear, investigations in the USA by Zavar et al (2023, 2016) recognise that land use change can lack formal or informal utility, with overgrown vegetation, or illegal dumping contributing to issues of blight. Sometimes, remaining residents may prefer basic naturalisation rather than ecosystem restoration (e.g. Sayerville, Table S1). This highlights that even with planned and zoned functions, such as ‘Open Space’, there is a broad continuum of utility and value to communities and ecosystems under ‘land use change’. Recognising the importance of planning for landscapes rather than sites, Binder et al. (2020) emphasise that the retreat effect is not bounded by the margins of withdrawal, impacting peripheral communities. Territorial margins may be further softened by transition from private property to alternative forms of tenure, including traditional tenure, or to no tenure at all. The reshaping of ‘dominion’ as a consequence of climate-driven geographical shifts is a recurring theme demanding reimagination of underpinning frameworks and concepts.

Importantly, not all retreats require significant land use change. Continued farming with altered living conditions means there is habitation change, but continuation of socio-ecological relations. This is an important function of origin sites where there are vital connections to local elements, highlighting that rather than ‘retreat’, a complex relationship represents adapting to changing conditions, with people co-evolving with the environment. In comparison with imposed colonial systems of planned retreat (Aiken and Mabon 2023) where land is held as private property, spatial reimagination can be a continual and ongoing process of relational co-evolution, highlighting a more fluid relationship and philosophy of people living as part of the environment.

This research also provides evidence that supports the argument that planned retreat is often detached from broader societal goals such as cultural or community cohesion, livelihoods, ecosystem health and housing (Mach and Siders 2021). To be transformative, planned retreat must attend to a more extensive set of goals than risk reduction or ecosystem restoration alone (ibid). This is particularly important when considering the publicly shared costs of retreat, and the future use, maintenance, and values of origin sites and their wider communities and environments. Demonstrating more strategic planning are areas purposefully redesigned with multiple land use functions to support a range of societal outcomes beyond the subject site. For example, several managed realignment projects revealed strategic multi-functionality, delivering saltmarsh restoration, open space land, blue–green infrastructure and protection measures with benefits *beyond* the site. Strategic functions may also be present in relation to individual sites, if a plan is in place to support wider spatial influence, such as planned demolition to reduce blight across a neighbourhood, or pollinator parks supporting a network of green infrastructure.

In some cases, there is evidence of progress towards land and seascape reimagination. This can be understood as designs for new socio-ecological land and seascapes which integrate various ecological, socio-cultural and political disciplines. We use the terms ‘landscape’ and ‘seascape’ to cut across territorial bounds that often constrain the reality of places as connected nodes within broader relational networks (Massey 2004) and ecosystems. Several cases demonstrated elements of networked land/seascape reimagination, for example, with integrated flood risk, water quality, habitat and community planning at the catchment scale (e.g. ‘Floodplains By Design’); comprehensive urban development incorporating planned retreat as a key facilitator in ‘making space for rivers’ and revitalising urban environments (e.g. ‘ENCLACE’, ‘Project Twin Streams’); and seascape revival, with waterfront acquisitions, seagrass restoration and living shorelines supporting habitat migration (e.g. Punta Gorda Adaptation Plan). A common feature of these is integrated, strategic and more relational planning which recognises inter-dependencies at multiple scales (Walsh et al. 2022). In some cases, such spatial reimagination will occur due to the relocation of an entire township (e.g. Princeville). Importantly, however, the case studies demonstrate the benefits of coordinated land use and adaptation policies where planned retreat is considered holistically. Projects working towards land/seascape reimagination often position retreat as one aspect of a broader plan, addressing strategic outcomes holistically.

In many instances, terrestrial landscapes will eventually exist underwater. This transition has several implications. Who governs these changing geographies and how, may determine whether this change is experienced as land loss, co-evolution or land–seascape reimagination. For example, Tulele Peisa is trying to navigate sea level rise and planned retreat in the Carteret islands, whilst ensuring cultural and livelihood connections remain. They have been advocating for the Carteret’s Islands to be declared a marine protected area, with provision for Indigenous management to preserve Indigenous knowledge and relations (UNDP 2016). ‘Sea Change’ raises questions of international law and governance, importantly whether people can maintain vital connections to islands and coasts, and whether established principles require rethinking in view of shifting geographies. Traditionally, the principle of ‘the land dominates the sea’ has applied, meaning that maritime baseline boundaries are ambulatory and move inwards with the retreating land. This has potential to significantly impact the spatial extent of national claims to maritime jurisdiction (Freestone and Schofield 2021). It is not clear whether, under the 1982 Law of the Sea Convention, the Exclusive Economic Zone (EEZ) baselines and limits will be recalculated when sea level changes affect the geographical realities of coastlines. Whilst there has been ‘considerable sympathy internationally’ for low-lying islands, ‘it remains to be seen whether the international community will countenance this potential evolution in the interpretation of the law of the sea as a new customary norm’ (Bernard et al. 2021, p. 18). This is a vital challenge for land–seascape relations. If island nations are subject to mobile baselines their maritime claims and socio-ecological relations may be under threat, no longer governed by legacy EEZ. Understanding and assessing the options for these areas may influence whether rising seas create sinking futures and relations, or solution spaces (White et al. 2023).

## Conclusion

This research emphasises the importance of thinking ‘beyond retreat’ at an early stage. The diversity of approaches has different legacies for communities either directly at risk, or in neighbouring areas, from ambitious restoration or reimagination schemes, to abandonment, and inequity. The cases provide evidence that narratives of ‘loss’ associated with planned retreats fail to capture land use dynamism and change that could be gained from considered spatial reconfiguration. Further, we strengthen concerns regarding the terminology of ‘retreat’; that ‘all is not lost’ or ‘left behind’. In some cases, land uses will remain intact, new functions may form, or others may be restored. Moving beyond retreat and loss narratives better highlights the diverse opportunities and contexts for adaptive spatial reorganisation that invite deeper theoretical, political and policy attention. The typology and dataset serve to highlight trends in practice, provide insights into optionality beyond retreat, support knowledge exchange, and demonstrate the need to match post-retreat aspirations with spatial context, ecosystem networks and community values.

Finally, we emphasise the importance of asking who and what are we reconfiguring space for? Risk management may provide the initial stimulus, but this technical perspective needs to be quickly expanded into different disciplines and collaborative practices to assist in envisioning and applying locally informed plans. There is a fundamental need for attention to justice and equity in how these spaces are planned, reimagined and governed. For example, who has a voice in spatial reconfiguration, what knowledge and value criteria are used, and what legacies are desired? Planned retreats pose crucial questions about who governs spaces that hold a multiplicity of relationships and meaning to people and ecosystems, and how these relations are altered. To address these questions, we call for a greater focus on spatial planning for land–seascapes beyond retreat, with attention to justice and relational spatial imaginaries.

## Supplementary Information

Below is the link to the electronic supplementary material.

Supplementary file1 (PDF 562 KB)

## Data Availability

Data used in this study is available in the sources cited in the Supplementary Information.

## References

[CR1] Aiken, G., and L. Mabon. 2023. Where next for managed retreat: Bringing in history, community and under-researched places. *Area*. 10.1111/area.12890.

[CR2] Ajibade, I., M. Sullivan, C. Lower, L. Yarina, and A. Reilly. 2022. Are managed retreat programs successful and just? A global mapping of success typologies, justice dimensions, and trade-offs. *Global Environmental Change*. 10.1016/j.gloenvcha.2022.102576.

[CR3] Bernard, L., M. Petterson, C. Schofield, and S. Kaye. 2021. Securing the limits of large ocean states in the pacific: defining baselines limits and boundaries amidst changing coastlines and sea level rise. *Geosciences*. 10.3390/geosciences11090394.

[CR4] Binder, S.B., L.A. Ritchie, R. Bender, A. Thiel, C.K. Baker, E. Badillo, S. Goodfellow, B. Kulp, et al. 2020. Limbo: The unintended consequences of home buyout programmes on peripheral communities. *Environmental Hazards* 19: 488–507. 10.1080/17477891.2020.1714537.

[CR5] Bronen, R. 2014. *Climate displacement in the United States: The case of Newtok village*. Alaska. In Land Solutions for Climate Displacement: Routledge.

[CR6] Brown, I. 2022. Do habitat compensation schemes to offset losses from sea level rise and coastal squeeze represent a robust climate change adaptation response? *Ocean and Coastal Management*. 10.1016/j.ocecoaman.2022.106072.

[CR7] Carter, J., J. Handley, T. Butlin, and S. Gill. 2017. Adapting cities to climate change: Exploring the flood risk management role of green infrastructure landscapes. *Journal of Environmental Planning and Management* 61: 1–18. 10.1080/09640568.2017.1355777.

[CR8] Daskalova, G.N., and J. Kamp. 2023. Abandoning land transforms biodiversity. *Science* 380: 581–583. 10.1126/science.adf1099. 37167371 10.1126/science.adf1099

[CR9] ELC. 2000. *European Landscape Convention*. Council of Europe. https://rm.coe.int/16807b6bc7

[CR10] Freestone, D., and C. Schofield. 2021. Sea level rise and archipelagic states: A preliminary risk assessment. *Ocean Yearbook Online* 35: 340–387. 10.1163/22116001_03501011.

[CR11] Funder, M., C. Mweemba, and I. Nyambe. 2018. The politics of climate change adaptation in development: Authority, resource control and state intervention in rural Zambia. *The Journal of Development Studies* 54: 30–46. 10.1080/00220388.2016.1277021.

[CR12] Galan, J., F. Galiana, D.J. Kotze, K. Lynch, D. Torreggiani, and B. Pedroli. 2023. Landscape adaptation to climate change: Local networks, social learning and co-creation processes for adaptive planning. *Global Environmental Change* 78: 102627. 10.1016/j.gloenvcha.2022.102627.

[CR13] Gonçalves, C., and P. Pinho. 2024. A manifesto for coastal landscape governance: Reframing the relationship between coastal and landscape governance. *Ambio* 53: 1454–1465. 10.1007/s13280-024-02040-5. 10.1007/s13280-024-02040-5 PMC11383885 38822969

[CR14] Hanna, C., R. Cretney, and I. White. 2022. Re-imagining relationships with space, place, and property: the story of mainstreaming managed retreats in Aotearoa-New Zealand. *Planning Theory and Practice* 23: 681–702. 10.1080/14649357.2022.2141845.

[CR15] Hanna, C., I. White, and B.C. Glavovic. 2021. Managed retreats by whom and how? Identifying and delineating governance modalities. *Climate Risk Management* 31: 100278. 10.1016/j.crm.2021.100278.

[CR16] Haughton, G., and I. White. 2018. Risky spaces: Creating, contesting and communicating lines on environmental hazard maps. *Transactions of the Institute of British Geographers* 43: 435–448. 10.1111/tran.12227.

[CR17] Hino, M., C.B. Field, and K.J. Mach. 2017. Managed retreat as a response to natural hazard risk. *Nature Climate Change* 7: 364–370. 10.1038/nclimate3252.

[CR18] Lawrence, J., J. Boston, R. Bell, S. Olufson, R. Kool, M. Hardcastle, and A. Stroombergen. 2020. Implementing pre-emptive managed retreat: Constraints and novel insights. *Current Climate Change Reports*. 10.1007/s40641-020-00161-z.

[CR19] Mach, K.J., and A.R. Siders. 2021. Reframing strategic, managed retreat for transformative climate adaptation. *Science* 372: 1294–1299. 10.1126/science.abh1894. 34140383 10.1126/science.abh1894

[CR20] Martin, P.C.M., P. Nunn, J. Leon, and N. Tindale. 2018. Responding to multiple climate-linked stressors in a remote island context: The example of Yadua Island, Fiji. *Climate Risk Management* 21: 7–15. 10.1016/j.crm.2018.04.003.

[CR21] Massey, D. 1993. *Power-geometry and a progressive sense of place*. In Mapping the Futures: Routledge.

[CR22] Massey, D. 2004. Geographies of responsibility. *Geografiska Annaler: Series B, Human Geography* 86: 5–18. 10.1111/j.0435-3684.2004.00150.x.

[CR23] Massey, D. 2006. Space, time and political responsibility in the midst of global inequality (Raum, Zeit und politische verantwortung inmitten weltweiter ungleichheiten). *Erdkunde* 60: 89–95.

[CR24] May, A., and D. Smart. 2003. Managed retreat of the Essex coast. *Geography Review* 17: 38–41.

[CR25] Ministry of Economy Republic of Fiji. 2018. *Fiji Planned Relocation Guidelines*. https://fijiclimatechangeportal.gov.fj/wp-content/uploads/2022/01/Planned-Relocation-Guidelines_Fiji.pdf

[CR26] Moher, D., A. Liberati, J. Tetzlaff, and D.G. Altman. 2009. Preferred reporting items for systematic reviews and meta-analyses: the PRISMA statement. *Physical Therapy* 89: 873–880. 10.1093/ptj/89.9.873. 19723669

[CR27] Moser, S. 2021. Waves of grief and anger: Communicating through the “end of the world” as we knew it. In I. Ajibade and A. Siders (Eds.), *Global Views on Climate Relocation and Social Justice: Navigating Retreat*. Routledge.

[CR28] Mossman, H.L., N. Pontee, K. Born, C. Hill, P.J. Lawrence, S. Rae, J. Scott, B. Serato, et al. 2022. Rapid carbon accumulation at a saltmarsh restored by managed realignment exceeded carbon emitted in direct site construction. *PLoS ONE*. 10.1371/journal.pone.0259033. 10.1371/journal.pone.0259033 PMC9710768 36449465

[CR29] O’Donnell, T. 2022. Managed retreat and planned retreat: A systematic literature review. *Philosophical Transactions of the Royal Society B: Biological Sciences* 377: 20210129. 10.1098/rstb.2021.0129. 10.1098/rstb.2021.0129 PMC9108935 35574844

[CR30] Siders, A., I. Ajibade, and D. Casagrande. 2021. Transformative potential of managed retreat as climate adaptation. *Current Opinion in Environmental Sustainability* 50: 272–280. 10.1016/j.cosust.2021.06.007.

[CR31] Siders, A., M. Hino, and K. Mach. 2019. The case for strategic and managed climate retreat. *Science* 365: 761–763. 10.1126/science.aax8346. 31439787 10.1126/science.aax8346

[CR32] Thomas, D. 2006. A general inductive approach for analyzing qualitative evaluation data. *American Journal of Evaluation* 27: 237–246.

[CR33] Tsang, M., and Stein, I. 2021. Losing ground: Rethinking land loss in the context of managed retreat. In I. Ajibade and A. Siders (Eds.), *Global Views on Climate Relocation and Social Justice: Navigating Retreat* (p. 324). Routledge.

[CR34] UNDP. 2016. Tulele Peisa, Papua New Guinea. Equator Initiative Case Study Series.

[CR35] van Rooij, S., W. Timmermans, O. Roosenschoon, S. Keesstra, M. Sterk, and B. Pedroli. 2021. Landscape-Based visions as powerful boundary objects in spatial planning: lessons from three Dutch projects. *Land* 10: 16. 10.3390/land10010016.

[CR36] Walsh, C., M. Lennon, M. Scott, and F. Tubridy. 2022. Spatial imaginaries in flood risk management: Insights from a managed retreat initiative in upper Bavaria. *Journal of Environmental Planning and Management*. 10.1080/09640568.2022.2082927.

[CR37] Wheeler, S.M. 2002. The new regionalism: key characteristics of an emerging movement. *Journal of the American Planning Association* 68: 267–278. 10.1080/01944360208976272.

[CR38] White, F.R., S.C. Urlich, and H.G. Rennie. 2023. Newly-claimed seascapes: Options for repurposing inundated areas. *Global Environmental Change Advances* 1: 100002.

[CR39] Zavar, E., A. Greer, S.B. Binder, and S. Niazi. 2023. The expression of visual culture on flood buyout landscapes, Harris County. *TX. Geojournal* 88: 5287–5304. 10.1007/s10708-023-10920-8.

[CR40] Zavar, E., and Hagelman, I. R. R. 2016. Land use change on U.S. floodplain buyout sites, 1990–2000. *Disaster Prevention and Management*, 25: 360–374. 10.1108/DPM-01-2016-0021

