# Supplementary material for: Beyond retreat: Land–seascape legacies of change and continuation
Source: Ambio. 2025 Mar 25;54(7):1199–212. doi: 10.1007/s13280-025-02142-8 (PMC12133652; doi:10.1007/s13280-025-02142-8)
Supplement: Supplementary file 1 — Supplementary file1 (PDF 562 KB) [file 13280_2025_2142_MOESM1_ESM.pdf]

Ambio Supplementary Information

This supplementary information has not been peer reviewed.

Title: Beyond Retreat: Land-seascape legacies of change and continuation

Tables S1, S2, S3,

and S1 references.

Table S1 to 'Beyond Retreat: Land-seascape legacies of change and continuation.

Country	Village/ City	# of households/ assets relocated or site area for managed realignment (ha)	Risk	Description	Functional codes	Coding detail	Sources
1. Zambia	Namapande Resettlement Scheme, Kazungula district	160	Riverine risks	Some people remained in-situ without state support in the event of future floods or drought. Transfer of the land title from customary title with chiefly authority to private deeds controlled by the State following resettlement. This resettlement scheme illustrates how climate adaptation interventions provide a means for the state to legitimise extension of control over customary land and production (Funder et al 2016).	*People remain in-situ	^Political climate change adaptation agenda ('green grabbing' of local resources (1))	1,2
2. Mozambique	Lower Zambezi - Various	Government attempt at resettling 56,000 displaced people	Riverine risks	Land has remained in agricultural use - strong cultural connections to the land remain. Government withdrew basic services in the origin areas and provided infrastructure in the new locations. People who could not purchase new land, ended up on marginal plots and therefore usually preferred going back to the lowlands or maintaining a double residence in the origin and resettlement areas. Whilst aimed at flood risk reduction, relocation also appears to be related to control and modernisation of Mozambicans (3). Government perspective of improved security and more efficient land use. Other local perspectives of harm to livelihoods, loss of assets and cultural identity. (3).	*Farming continues (relational co-evolution)<br>*People remain in-situ	^Withdrawal of services<br>^Political climate change adaptation agenda<br>^Livelihood and socio-cultural concerns	2,3
3. Malawi	Jombo, Chikwawa district	60	Riverine risks	Around 60 households in the village resettled. Many, along with the village head, preferred to stay in their previous location. Land has remained in agricultural use	*Farming continues (relational co-evolution)<br>*People remain in-situ		2,4
4. China	Massive Southern Shaanxi Migration Program, Chengguan, Shangnan County	600000 household aim	Multiple	Land has remained in agricultural use during transition phase	*Farming continues (relational co-evolution)		2,5
5. China	Yangtze River basin	2400000	Multiple	Wetlands restored and reconnected to the river	*Floodplain restoration		6,7
6. China	Qinghai, Madoi County	585	Degraded grasslands (with climate change influence)	Grazing ban to restore the ecological environment	*Ecological restoration	*Regulation - Grazing ban*	2,8
7. India	Chennai, Tamil Nadu	1392	Coastal risks	Fishing communities were relocated, affecting Indigenous rights to coastal land, yet tourism and industry infrastructure remained intact. In some coastal villages, the government sold coastal land to private developers after displacing the fishing communities in the name of conservation. Fishing communities were unhappy with the government's decision to move them away from the coast - some continued living in temporary shelters or moved back into their damaged homes. Anna Nagar villagers filed a petition with the High Court and received a stay on the eviction. Not all efforts to resist relocation were successful. People relocated to Kargil Nagar, for example, tried to move back to the coast, but the government forced them back to their relocated homes. Government planting of Casuarina tree as a bioshield along the Kariakal and	Multiple uses	*Redevelopment<br>*People remain in-situ<br>*Blue-green infrastructure<br>^Plantation choice concerns<br>^Livelihood concerns	2,9–12

				Nagapattinam coast. Fishing communities claim to have voiced their concerns to the government, including their preference for mango and coconut trees, which have a greater chance of survival. Fishing communities were strongly opposed to the plantations and in some instances uprooted the saplings, which compromised their access to boats and the sea. [9]			
8. India	Relocation from Kamgar Putala slum to a housing society at Hadapsar, Pune	152	Riverine risks	Old site now protected by a flood retaining wall and undergoing a government slum upgrading scheme. Uncoordinated relocation and protection planning.	*Redevelopment		12,13
9. Iran	Balklor and Jamal-Abad village residents were relocated to Ab-Bar village, Zanjan	~200	Geological-Earthquake	Land has remained in agricultural use - households that wanted to continue with animal husbandry have returned to their original location or commute between their former and current locations.	*Farming continues (relational co-evolution)<br>*People remain in-situ		12,14
10. Indonesia	Solo/Surakarta, Central Java	~1000	Riverine risks	No evidence of planning for retreated site and those remaining in-situ and at risk	*People remain in-situ		15,16
11. Indonesia	Aceh Province	Unknown	Coastal risks	Buffer/No-build zones were not strictly enforced meaning there has been reoccupation of the zones e.g. in some places fishing communities are still allowed to live within these zones	*Reoccupation		12,17,18
12. Philippines	Cagayan De Oro, Northern Mindanao	6,421	Riverine risks	Buffer/ No-build zone enforced by Local Government Unit within 3 metre easements of rivers to prohibit urban use.	*Open Space		2,19
13. Philippines	Cagayan de Oro, Northern Mindanao - Indahag	590	Riverine risks	Buffer/No-build zones were not strictly enforced meaning there has been reoccupation of the zones	*Reoccupation		2,20
14. Philippines	Cagayan de Oro, Northern Mindanao - Macapaya	408	Riverine risks	Buffer/No-build zones were not strictly enforced meaning there has been reoccupation of the zones	*Reoccupation		2,20
15. Philippines	Cagayan de Oro, Northern Mindanao - Ecoville	518	Riverine risks	Buffer/No-build zones were not strictly enforced meaning there has been reoccupation of the zones	*Reoccupation		2,20
16. Philippines	Tacloban, Region VIII (Eastern Visayas)	10,000	Coastal risks	Buffer/No-build zone enforced 40m from shore	*Open Space		21–23
17. Philippines	Leyte and Samar	19,330 houses have been constructed for those displaced by the no-build zone as of March 2016. 205,000 total houses are planned	Typhoon Haiyan	Buffer/No-build zone - But many internally displaced persons chose to return to their former communities and reconstruct their homes using whatever storm debris they could salvage, despite the prohibition on rebuilding.	*Reoccupation		24
18. Sri Lanka	Nationwide	32-33,000	Coastal risks	Buffer/No-build zone – not strictly enforced. Buffer zones were originally 100-200m but were reduced to 25-50 (southern districts) and 50-100m (northeast). Within the buffer zone any construction or reconstruction was prohibited except for port structures, historical monuments and tourist centres opening the space for a 'disaster capitalism' to develop. Many people refused to move. Tsunami caused pollution of the coastal strip (solid waste, debris, and seawater contamination), and impacts on coastal ecosystems. Rehabilitation efforts included the setbacks, reconstructing protective structures, rehabilitating sand dunes, mangrove planting and establishing green belts along the coastline.	Multiple uses	*People remain in-situ<br>*Redevelopment (tourism, port)<br>*Protection measures<br>*Coastal restoration (dune rehabilitation, mangrove, and green belt planting)<br>*Open Space	2,10,25–28

19.	Taiwan	Rinari	483	Geological-Landslide	Heritage preservation in old site much later than when resettlement was undertaken. – original resettlement process did not pay attention to sense of place or heritage.	*Heritage preservation		2,29
20.	Japan	Tohoku - Various	~500,000 people displaced (Pinter)	Tsunami	Reconstruction of coastal dikes (including significant additions and enhancements – 'The Great Wall of Japan'), establishing hazardous zones, regenerating coastal forests and igune for disaster risk reduction, building embankments along the Teizan Canal and other rivers, building evacuation hills and towers, constructing elevated roads, memorial facilities, food frontier project to revitalise agriculture (remove rubble and salt, restore drainage and pumps).	Multiple uses	*Protection and accommodation measures<br>*Coastal restoration<br>*Open Space<br>*Heritage preservation - Memorial facilities<br>*Farming continues (*Land rehabilitation)*<br>*^Tourism*	30,31
21.	Malaysia	Sungai Lurus, Western Johor		Coastal risks	Abandonment of defences and agricultural land to mangroves, forced by erosion.	*Sea Change		32
22.	Vietnam	Nam Ha, Hai Hau District		Coastal risks	Abandonment of coastal protection, land is lost to the sea. Not specified whether removing existing residential structures	*Sea Change		33
23.	Nepal	Dhye, Upper Mustang	10	Geological-Landslide	Gradual abandonment of village for new location	*Abandonment		2,34
24.	Guatemala	Panabaj and Tz'anchaj, Santiago Atitlán, Department of Sololá	915	Geological - Debris flow	Buffer zone/No-build regulations - the government prohibited investments by public agencies and international organizations in the high-risk areas	*Open Space		12,35
25.	United States	El Caño Martín Peña: San Juan, Puerto Rico	600	Riverine, ecological and health risks and wider risks to catchment e.g., airport	Comprehensive Development and Land Use Plan for new stormwater, wastewater and potable water distribution systems, acquisition and demolition of structures and relocation of eligible occupants. Caño Martín Peña Ecosystem Restoration Project, dredging the Caño to restore the flow of water between the San Juan Bay and the eastern Estuary, help healthy mangroves and coral reefs flourish, increasing biodiversity and essential fish habitats, creating a navigable Caño with new waterfronts, reconnecting points of historical, cultural, and tourist interest throughout the Metropolitan Area. Health conditions for over 15,000+ persons affected by frequent floods with raw sewage and mosquito transmitted diseases (dengue fever, zika, chikungunya) are expected to improve. Partial estimates point that the project will benefit the economy by $587 million, whereas avoided costs include estimated losses of $700 + million during a 100-year recurrence flood event. >4,000 jobs created during construction of the ecosystem restoration project. Residents are resettled within the same community via flood-adapted infill.	Multiple uses – Networked (strategic, integrated landscape planning)	*Coastal restoration (reconnecting the estuary, mangrove and coral reef restoration)<br>*Redevelopment – (infrastructure upgrade, new waterfronts reconnecting tourism, heritage, culture interests, adaptive urban infill)<br>^Job creation<br>^ Health and safety<br>^ River habitat<br>^Improved resilience of critical infrastructure (airport)	2,23,36–39
26.	Brazil	Ilha do Cardoso, São Paulo	~10	Coastal risks	Land nearly lost to the sea.	*Sea Change		2,40

27.	Brazil	Tietê River Basin Stream Canalization Program (PROCAV) São Paulo	7,722 families (2,585: 1987-1994, and 5,137: 1995-2007)	Riverine risks	Stream canals and dams developed alongside relocation and prohibition of use of vacant land. Establishing and protecting green areas to reduce erosion and sedimentation, reduce stream pollution by eliminating the discharge of solid wastes and untreated sewage, improve health and housing conditions of the families affected via urban development and improvement of transportation by building roads parallel to the canalized sections. However, lack of networked, integrated approach – channelisation created suffocation of rivers, creating a profoundly impermeable landscape marked by extensive flooding over time, reflecting infrastructural imaginaries. Recent plans to make more space for rivers.	Multiple uses	*Open Space<br>*Redevelopment (infrastructure, transport, housing, sewerage)<br>^Health and safety benefits	12,35,41
28.	Brazil	Rio Grande do Sul, Osório	Infrastructure - Road	Coastal risks	Removal of road, installation of sand fences and control of pedestrian accesses to enable restoration of foredune at Osório and protection of dune vegetation.	*Coastal restoration	*Open Space	42
29.	Colombia	Buenaventura, San Juan River Delta	N/A	Riverine risks	Loss of coastal lands and islands has meant an increase in timber exploitation.	*Sea Change		2,43
30.	Colombia	Amanzaguapos /Pueblo Nuevo	N/A	Coastal risks	Land lost to the sea.	*Sea Change		44
31.	Colombia	Nueva Esperanza, Rafael Uribe, Bogotá	1074	Geological - Land instability, erosion + landfill and wastewater discharge issues	Rehabilitation and Restoration planting, incorporated into PEDEN Ecological Park	*Ecological restoration	*Open Space	12,35
32.	Argentina	Various	11,911	Riverine risks	Open space	*Open space		12,35
33.	Peru	San Martín	30 (150 farmers)	Riverine risks	'Village 1' - Abandonment of structures and land swap with local agribusiness	*Commercial		2,45
34.	United States	Yup'ik, Newtok, Alaska		Coastal risks	Transition phase - Newtok still needs more funds to complete the relocation. Until then, the current site must be maintained to accommodate residents who have not yet relocated. However, the village is no longer eligible for capital funding for improvements to infrastructure due to the transition phase. Contamination and pollution concerns with river flooding and erosion impacting raw sewerage ponds and landfill site. Buildings are being demolished as erosion continues to encroach upon the old village. In 2022, it was stated that for the remaining residents to relocate to Mertarvik, further funding was needed for 26- 28 homes.	*Sea Change and transition phase<br>*Demolition<br>^Contamination concerns	Permafrost thawing	46–48
35.	Canada	Gatineau, Quebec	185	Riverine risks	Structures have been demolished and properties have been officially transferred to the City of Gatineau. Community engagement and visioning events focused on post-retreat community redevelopment including designing the post-flood, post-retreat landscape for community benefit and liveability. Neighbourhood revitalisation projects on vacant lots. Future plans for urban gardens, commemorative benches, arboretum, community walkway and parks in vacant lots. Typologies in Master Plan: Nature lots - pollinator lots, meadows, and wooded areas; Nourishing lots - fruit trees, urban farming and greenhouse lots- Gathering lots: community tables, places for play (basketball courts), amphitheatre, community art and dog parks; Lots onshore - river terraces, basin drainage lots and bridging lots. Sponge sets: Consisting of lots that provide ecological opportunities. Concerns from some about checkerboarding and impacts on neighbouring/ buyout excluded properties.	Multiple uses	*Blue-green infrastructure (sponge sets, drainage, and bridging lots)<br>*Open space (urban gardens, commemorative benches, arboretum, community walkway and parks, nourishing, gathering, river terraces)<br>^Food production<br>^Concerns regarding checkerboarding	2,49–52

36.	Canada	Beachwood Estates, High River, Alberta	31	Riverine risks	Basic site rehabilitation – Structures, paths and infrastructure have been removed from the sites and basic naturalization undertaken, which will allow the river to flow more freely and support flood mitigation for the wider Town.	*Open Space (basic)	*Blue-green infrastructure - Naturalisation of floodway<br>^Risk reduction (wider township)	2,52–54
37.	Canada	Wallaceville High River, Alberta	94	Riverine risks	Basic site rehabilitation – Structures, paths and infrastructure have been removed from the sites and basic naturalization undertaken, which will allow the river to flow more freely and support flood mitigation for the wider Town.	*Open Space (basic)	*Blue-green infrastructure - Naturalisation of floodway<br>^Risk reduction	2,52–54
38.	Canada	Aulac, New Brunswick	16.5ha	Coastal risks	Saltmarsh restoration	*Coastal restoration – salt marsh	*Blue-green infrastructure<br>*Protection measure (remains)	2,55
39.	Canada	Onslow-North River Dyke Realignment and Tidal Wetland Restoration Project - Truro-Onslow Dyke project, Nova Scotia	92ha	Riverine risks	Saltmarsh and habitat restoration. Remnants of the old dyke remaining. Archaeological and 250-year-old cemetery damage avoidance. Engagement concerns that site would become a mosquito breeding ground, was subsequently addressed through a management plan. Habitat credits banked for NSTIR infrastructure projects. Benefits of water filtration, carbon sequestration, biodiversity and pollination services, wildlife viewing and restored fish access, reduce flood risk for the town of Truro and reduce dyke maintenance costs.	Multiple uses	*Coastal restoration – tidal wetland<br>* Heritage preservation<br>*Open Space<br>^Habitat restoration – restored fish access<br>^Risk reduction (wider township)<br>^Carbon accumulation<br>^Biodiversity services<br>^Water filtration<br>^Reduced dyke maintenance costs	2,52,56–58
40.	Canada	Leamington, Ontario	Road realignment	Coastal risks	500 m portion of a bluff top road known as Bluff Line was realigned approximately 75 m inland due to nearby rapid bluff erosion of 1.4 m/year	*Open Space (Lake)		2,52
41.	United States	Valmeyer, Illinois	120	Riverine risks	Open Space - for recreation and farming purposes. Rows of corn now earn the town about $25,000 a year. 50 residents stayed behind in the floodplain. One remnant of the original town is Borsch Park which remains amongst those agricultural fields. In Borsch Park, a plaque dedicates the pavilion to a local resident and references the flood and subsequent buyout [59]	Multiple uses	*Open Space - Corn farming and recreation<br>*People remain in-situ<br>*Heritage preservation - Plaque in Borsch Park<br>^Economic benefit to town from horticulture	2,50,59–62
42.	United States	Cedar Rapids, Iowa	1356 properties (including businesses)	Riverine risks	Managed retreat was part of a larger recovery and mitigation strategy that partitioned the floodplain into three management areas: 1. Construction Area for structural flood management system, 2. Greenway Area, 3. Neighborhood Revitalization Area. River Corridor Redevelopment Plan combined levees, removable floodwalls, open space, and neighbourhood revitalisation. Some of the proposed amenities are picnic areas, soccer fields, skate and dog parks, a botanical garden, disc golf course, fishing pier, canoe/kayak launch, and open-air pavilions. After the installation of the flood management system in the neighbourhood revitalisation zone, the acquisition of these properties is expected to contribute to community development as they will be redeveloped, with priority given to buyers with incomes below the area median value.	Multiple uses – networked – strategic planning of floodplain	*Open Space *(soccer fields, botanical garden, disc golf, kayak launch etc)*<br>*Protection measures<br>*Redevelopment (Housing development and affordability)	2,63,64
43.	United States	Ames, Cherokee, Des Moines, Louisa County, Wapello, Iowa	1400	Riverine risks	Floodwater retention land and recreation space	Multiple uses	* Open Space – Recreation<br>*Blue-green infrastructure - Floodwater retention land	64
44.	United States	Resilient Edgemere - Queens Build it Back Buyout Program, New York	65	Coastal risks	The Resilient Edgemere Community Plan sets out a long-term vision to achieve a more resilient neighborhood with improved housing, transportation access, and local amenities. This plan was created alongside a citywide housing recovery program which included a “land swap” pilot project to	Multiple uses – networked – (strategic,	*Open Space (passive recreation)<br>*Protection measures	2,65–68

				provide buyout and relocation assistance to residents within a "Hazard Mitigation Zone", The City acquires the homeowner's damaged property and will use the land to develop future coastal protection structures, open space use, and improve waterfront access. Damaged structures (including on vacant and abandoned properties) will be demolished. The neighborhood plan aligns resilience planning and disaster recovery with New York City's affordable housing and climate adaptation goals. Resilient Edgemere provides an example of how a land swap can enable people to relocate nearby, maintaining community cohesion and connections with comprehensive planning.	integrated landscape planning)	*Redevelopment (transport, housing, and amenity planning)<br>^Improved urban waterfront access	
45. United States	Floodplains by Design: Rural Washington	*2000 homes and structures	Integrated floodplain management for fish habitat, river systems and flood mitigation	Floodplain restoration and reconnection, integrated catchment management at the landscape scale: "Floodplains by Design works to reduce flood risk, restore habitat, improve water quality, support agriculture and enhance recreation along Washington's rivers. By transforming how floodplains are managed on a landscape scale, Floodplains by Design supports thriving communities and a healthy environment. The heart of this approach is the idea that the most complex problems are solved by helping people work together. Trust is built through structured conversations that give voice to the values of each interest group, and coordinating investments toward local solutions." [69] Projects must support integrating flood hazard reduction with ecological preservation and restoration but can also support other community needs, provided they are part of a larger strategy. Some examples include agriculture preservation, water quality improvements, increased recreational opportunities. floodplain reconnections, fish and wildlife habitat restoration, floodway planting.	Multiple uses – networked (strategic, integrated landscape planning)	*Floodplain restoration and reconnection<br>*Open Space<br>*Farming continues<br>*^Habitat restoration*	69–72
46. United States	Soldiers Grove, Wisconsin	10	Riverine risks	Open space - Riverside Park with picnic areas, tennis courts and campgrounds. Public participation in planning of origin site.	*Open Space	Riverside Park (picnic areas, tennis courts and campgrounds)	2,64,73–76
47. United States	Devil's Slide, California	N/A	Geological - Landslide	Open space - Highway 1 used to create a 1.3-mile hiking and bike trail, connecting to the larger network of trails in the area	*Open Space	Hiking and bike trail	2,77
48. United States	Dorchester County, Maryland	410 acres	Coastal risks	Restoration and marsh migration on an *ecosystem scale* that necessitates public and private land acquisitions: Strategic restoration to improve tidal marsh health and functioning (including improving water quality), strategic conservation in priority marsh migration corridors and actions to support the transition of uplands to marsh. Visitors to Blackwater will be able to enjoy birdwatching.	Multiple uses – networked (ecosystem-based scale with strategic interventions)	*Coastal restoration<br>**Open Space*<br>*^Water quality improvement - planting of transition crops*<br>*^Ecosystem migration space*	2,78
49. United States	Foster Floodplain Project: Portland, Oregan	60	Riverine risks	Integrated planning supporting the Portland Watershed Management Plan, Johnson Creek Restoration Plan, to improve water quality, enhance habitat, and prevent damage from floods. It also supports Portland Parks & Recreation's Natural Area Acquisition Strategy. Floodplain and creek restoration, green infrastructure, and accessible recreation improvements (new flood storage, removed infrastructure and soil, created two backwater channels to provide resting places for fish during high flows, enhanced two ponds as habitat for sensitive red-legged frogs and Northwestern salamanders, used downed trees in brush piles for upland habitat, and planted vegetation, added an ADA-accessible trail and pedestrian bridge to view Johnson Creek and wildlife. Compensation was given to homeowners in the form of buyouts. After 11 years all residents had left the neighbourhood.	Multiple uses – networked (strategic, integrated planning at the catchment scale)	*Floodplain & creek restoration<br>*Open Space<br>*Blue-Green infrastructure (flood storage)<br>*^Accessibility improvements*<br>*^Habitat enhancements*	2,79
50. United States	Punta Gorda, Florida	N/A	Coastal risks	Land acquisitions, relocation of buildings and infrastructure, living shorelines (increasing sea grass acreage by 58%) that can act as a flood buffer and facilitate the inland migration of coastal habitats, restoration of vacated space to their natural conditions and creating public parks. Historic buildings are prioritized for relocation to help preserve cultural heritage. Voluntary annexation policy to enable the City to acquire safer land to potentially relocate development and infrastructure locally, in addition to broader land	Multiple uses – networked (strategic, integrated land-seascape planning)	*Coastal restoration<br>*Blue-green infrastructure – living shoreline flood buffer<br>*Open space – parks<br>*Heritage preservation (relocation)	2,23,78,80

					use strategies and regulations to enhance the city's long-term resiliency (such as reviewing land-use plans according to shifts in development patterns due to climate change). Punta Gorda provides an example of how adaptation plans can be used to inform land-use decisions and institutionalized through other local plans to achieve a broader vision, in this case, commitment to maintain a liveable, historic city while preparing for climate change.		*^Coastal ecosystem migration facilitation*	
51.	United States	Charlotte, North Carolina	700	Coastal risks	Open space (community gardens, trails, floodplain restoration and some unmaintained/removed streets and utilities, and some allowance for older people to remain in situ - CMSS also works with community members to design and realize a vision for each large-scale bought-out area once all homes are purchased and demolished.<br><br>Leasebacks for older residents or people who need more time to plan their relocation, and orphan parcel leases, where a nearby property owner is willing to maintain a bought-out property in exchange for exclusive use of it. Lessees provide in-kind services, like yard maintenance, in exchange for the use of a property and are not charged any monetary rent. Leaseback arrangements support increased flexibility in buyouts. Although the CMSS program has used leasebacks in a small number of cases, it has provided additional benefits to individuals and the larger community by enhancing community cohesiveness, offsetting acquisition costs, reducing property maintenance costs, and addressing the specific needs of property owners. ~ 85 percent of property owners within priority flood-risk reduction areas have elected to participate in the Buyout Program.	Multiple uses	*Open Space (community gardens, greenway trails and paths and some unmaintained areas)<br>*Floodplain restoration<br>*Residential Leasebacks - for older people and neighbourhood maintenance and use leases<br>*People remain in-situ	2,78,81
52.	United States	Greenville, North Carolina	491	Coastal risks	Open space – Land Reuse Plan states the areas may be leased out to adjoining property owners for increasing yard space, gardening, parking, etc., or used for recreational, and wetland management uses including parks, nature reserves, cultivation, grazing, and camping.	*Open Space		2,73,82
53.	United States	Kinston, North Carolina	775	Coastal risks	Open space - Infrastructure removed and recreational spaces and parks created. Attempts at more networked approaches (Green infrastructure plan, Green Open Space Plan, Plan Kinston Land Suitability Analysis and Kinston Waterfront Now!) but with limited implementation. A need for comprehensive land use planning, including adaptation. Open Space area includes the decommissioned wastewater treatment plant and a former landfill.	*Open Space – some attempts at networked planning	Parks and Recreation, Greenway Corridor and trails, educational forest/gardens,<br>^Former landfill and decommissioned wastewater treatment plant	2,64,83–85
54.	United States	Grand Forks, North Dakota	802	Riverine risks	Voluntary acquisition of properties, relocation of salvageable homes out of the flood plain and demolition of unsafe structures. New dikes and unique regional greenway system within the floodway of two rivers, creating a large 2200-acre riverfront greenway hosting two golf courses, three-disc golf courses, trails, shore bank fishing sites, campground, four boat ramps, and riverbank stabilization (riparian planting) and wildflower restoration. Along with flood protection measures, the greenway has proven to reduce flood risk to the city and helped to revive its downtown areas due to the improved flood safety. In addition, the Greenway, as one of Grand Forks' main amenities for recreation and tourism, helped anchor new development in the city's downtown, influencing the city landscape significantly.	Multiple uses – networked (strategic, integrated landscape planning)	*Open Space – greenway/ recreational park (golf course, trails, fishing sites, boat ramps, campground)<br>*Blue-green infrastructure (riparian planting)<br>*Ecological restoration – riparian planting and native wildflowers<br>*Protection measures (dikes)<br>^Urban revitalisation – greenway acting as anchor for investment<br>^Risk reduction to city from protection measure and BGI	2,46,64,86–88
55.	United States	Woodbridge Township, New Jersey	142	Riverine risks	Vacated properties are demolished and restored to natural wetlands and recreational amenities. Approach is to buyout contiguous lots ideal for restoration and management. Additional approach to purchase abandoned properties foreclosed by banks, maximising the open space conversions. In	Multiple uses – networked (strategic,	*Floodplain restoration<br>*Open Space – recreational/ community assets	2,78,89

				Woodbridge Township, the state and township are partnering with The Land Conservancy of New Jersey and Rutgers University to design an ecosystem-based flood buffer with passive recreational amenities for residents, like trails and a kayak launch, that can also become community assets. Woodbridge is working across sectors and agencies (e.g. community development, floodplain regulation, natural resources and emergency management, and land use and zoning) to utilize integrated buyouts for community redevelopment and resilience.	integrated landscape planning)	*Blue-green infrastructure (vegetative flood buffer)<br>^Blight reduction (purchasing abandoned properties too)	
56.	United States	Sayerville and South River, New Jersey	272	Riverine risks	*As of 2016, 20 homeowners who were eligible to participate in the buyout chose not to do so, the land procured in the buyouts currently is used as open space. Vacant lots sit largely unmanaged or unused for wildlife or recreation. The state wants the land to revert to its natural state, but the remaining residents prefer that the lots are grassed and mowed. The sidewalks bordering the lots and the street are still present, overgrown with weeds and almost unpassable.	*Open Space - basic<br>*People remain in-situ		90,91
57.	United States	Gonzales, Louisiana	41	Riverine risks	In 2020, the Silverleaf buyout program was complete and 41 property owners had relocated. Between 2020-2022, the area had flooded at least five times. The demolition of structures and environmental restoration work is complete. Over 2,000 native trees have been planted on the now city-owned land.	*Floodplain restoration	*Open Space	46,64,92
58.	United States	Project Home Again: New Orleans, Louisiana	101	Hurricane	Land swap program focussing on relocating neighbourhoods (rather than individual property owners). End use = Redevelopment, Private ownership, and Open space - for flood retention, environmental, and community benefits. Additional programme redevelopment partnership with NORA, the city's redevelopment authority, to coordinate its redevelopment strategy with the city's rebuilding priorities. "PHA's disposition strategies included donating properties to other nonprofits, selling properties at a discounted price to abutting homeowners, selling lots to small local developers or individuals, and leasing properties to individuals or organizations as community gardens." [93]	Multiple uses	*Blue-green infrastructure (flood retention)<br>*Open Space – environmental or community based<br>*Residential Amalgamations (neighbouring lots amalgamated)<br>*Redevelopment	46,93,94
59.	United States	Road Home Programme: New Orleans, Louisiana	5000	Hurricane	Redevelopment, private ownership, and open space (green space or water retention and detention uses). The City of New Orleans's Lot Next Door Ordinance gave homeowners living to the right or left of vacant properties a limited right of first refusal to purchase the property. The Lot Next Door Program promoted redeveloping adjacent properties as green space with a discount on the price if investment in greening and fencing occurred. The program provided relief to residents who could relocate, but there was a missed opportunity to achieve a transformed city and reduce future flood risk for those who remained.	Multiple uses – but missed opportunity to achieve a transformed city	*Redevelopment<br>*Open Space<br>*Blue-green infrastructure (water retention/detention)<br>*Residential Amalgamations (neighbouring lots amalgamated 'greened')<br>*People remain in-situ	91,95
60.	United States	Mastic Beach, Long Island, New York	7 NY Rising Deed restricted, plus 106 parcels for acquisition in 2020	Coastal risks	Strategic acquisitions to enable floodplain restoration, flood risk mitigation, and marsh migration to protect the low-lying coastal areas of the town that are most likely to flood during extreme high tides, storms, and hurricanes. The conceptual restoration plan restores degraded lands to native wetland and upland habitat and restores hydrologic connectivity to protect the community. At the same time, it enhances and reconnects fish and wildlife habitat, and creates space for marsh migration as a coastal resilience and climate adaptation strategy. "Considerations assessed in the restoration framework focused on maximizing the synergies between landscape complexity and connectivity, improvements to fish and wildlife habitat, and adding surface roughness to the land surface during storm events to aid in protecting upland communities from dynamic wave runup energy. Aspects of community recreation and public access were also considered in the restoration framework discussions."[96]	Multiple uses – networked (strategic, integrated landscape planning)	*Floodplain restoration<br>*^Habitat reconnection and enhancement*<br>*Blue-green infrastructure (storm and tidal buffer)<br>*Open Space<br>^Ecosystem migration space	91,96
61.	United States	Wayne, Hoffman Grove	96	Riverine risks	Given the patchwork nature of the buyout, it has been difficult to convert lands into forest, wetlands, or recreational areas. In some areas, including	*Open Space – basic		91,97

		neighbourhood, New Jersey			Hoffman Grove, where several acquired homes are still standing, public use of the acquired properties is limited…As in many other communities, however, the patchwork nature of the buyout limits the use of the acquired lands. Houses are interspersed among vacant lots. The buyout seldom comprises a sufficient number of contiguous properties to create parkland or wildlife habitat. However, over time, Wayne could purchase many, if not all, of the remaining properties in its flood hazard areas. [97] Eventually, the aim is to return the entirety of the Hoffman Grove neighborhood to floodplain.	*People remain in-situ (limiting land use)		
62.	United States	Oakwood Beach, Graham Beach and Ocean Breeze, Staten Island, New York	299	Coastal risks	Structures demolished. Restoration of natural flood and coastal buffers (maritime forests, tide gates, tidal wetlands, breakwater reefs, and earthen levees). Portions of the land have been converted to hiking trails, walkways, and wildlife observation areas, some properties may also be converted to community assets, such as sports fields. Concerns over poor maintenance of buyout area and impacts on peripheral communities. A small number of residents chose not to participate in the buyout were still living in the area.	Multiple uses	*Coastal restoration (forests, wetlands)<br>*Blue-green infrastructure (maritime forests, tidal wetlands, breakwater reefs)<br>*Protection measures (tide gates and earthen levees)<br>*Open Space (trails, wildlife observation areas, sports fields)<br>*People remain in-situ	2,78,91,98
63.	United States	Shawneetown, Illinois	?	Riverine risks	Most of the town moved to 'New Shawneetown.' Around 400 people remained. Buildings abandoned – 'ghost town'	*Abandonment – ghost town<br>*People remain in-situ		99,100
64.	United States	Leavenworth, Illinois	?	Riverine risks	Town relocated but reoccupation occurred due to lack of restrictions and private ownership - Construction of over 40 structures on the floodplain has added flood risk back to the old town	*Reoccupation		99
65.	United States	Pattonsburg, Missouri	271	Riverine risks	A total of 271 buyouts were completed. Churches and city buildings did not receive funds to move. The old town, is now disincorporated, still houses a few people who decided not to move… Universal Studios, used the bleak, abandoned town in 1999 to film Ang Lee's "Ride With the Devil."	*Abandonment – ghost town<br>*People remain in-situ		99,101
66.	United States	Rhineland, Missouri		Riverine risks	Landswap - Homes were hoisted by movers and rolled up the hill, one by one. Local businesses severely damaged by the flood also had strong community ties and business owners elected to remain with the village. A side of the bluff was excavated for new construction to make the town accessible to traffic along Highway 94 and close to the interstate Katy Trail. Eight businesses were rebuilt against the bluff, many feet below the hilltop community but above the floodplain. The community is now over 100 feet above the Missouri River.	*Open Space (community gardens, baseball park)		99,102
67.	United States	Wakenda, Missouri	43	Riverine risks	The land use of the former town is primarily agricultural - leased to a local farmer. Around the community designed granite monument/memorial, the U.S. Forest Service planted 30 trees to study the impact of flooding on local species.	Multiple uses	*Open Space – farming lease<br>*Heritage preservation – memorial	59
68.	United States	Hartsburg, Missouri	3	Riverine risks	The former private properties now offer a parking lot adjacent to the church and a small park with basketball hoops. Commemorative sign details the 1903, 1951, and 1993 floods.	Multiple uses	*Open Space (park and parking lot)<br>*Heritage preservation - Commemorative sign.	59
69.	United States	Odanah, Wisconsin	?	Riverine risks	Township relocation. All that remains in "Old Odanah" is the Catholic church next to the pow-wow grounds.	*Heritage preservation –pow-		99,103

						wow grounds and Church		
70.	United States	Grafton, Illinois	100	Riverine risks	Open space	*Open Space		99
71.	United States	English, Indiana	100 structures removed	Riverine risks	Golf course	*Commercial	Golf Course	99
72.	United States	Silex, Missouri	73	Riverine risks	Businesses, churches, and municipal buildings (except the school) remain in the floodplain	*People remain in-situ		99
73.	United States	Surfer's Point, Ventura County, California	Relocating bike path and parking lot	Coastal risks	Coastal restoration - dunes and vegetation in some areas	*Coastal restoration	Dunes and vegetation<br>*Open Space	104–106
74.	United States	Pacifica - San Mateo County, California	2	Coastal risks	Dune reconstruction and beach and estuary restoration	*Coastal restoration	Dunes, beach, and estuary<br>*Open space	104,106
75.	United States	Marina, Monterey County, California	Industry	Coastal risks	Community pressure successfully resulted in an agreement with Cemex to phase out sand removal by the end of 2020	*Open Space (beach)	Industry activity withdrawal	104
76.	United States	Princeville, North Carolina	?	Riverine risks	Comprehensive planning of the township to manage vacant sites, improve floodgates and infrastructure, protect heritage, and revitalise the town for heritage-based tourism.	Multiple uses – networked (strategic, integrated landscape planning)	*Protection measures<br>*Heritage preservation and tourism<br>*Redevelopment - Comprehensive planning for a connected system of educational, recreational, and environmental amenities	107
77.	United States	Nashville, Tennessee	56 properties (1977-2009). 246 properties (2010-2016)	Riverine risks	Open space - 43% of vacant lots are mowed grass, with the remainder of the area being naturally vegetated. Tree planting program is in place to combat tree canopy loss from urban development. 10,000 trees planted with high water retention capacity on bought-out parcels. "During rainfall events such as a 100-year storm (1% ACF event), one mid-sized tree may provide about 50 gallons (0.19 cubic meters) of stormwater retention" As of 2016, the residents of 48 properties declined to participate in the buyout and continue to reside in their repetitive flood loss homes.	Multiple uses	*Open Space - Mowed grass and tree plantings<br>*Blue-green infrastructure (water retention trees)<br>*People remain in-situ	108
78.	United States	Tulsa, Oklahoma	900	Riverine risks	Cleared floodplains were used for open space parks and trails, flood control works (small fish-stocked lakes were created from stormwater detention facilities) and urban wildlife habitat enclaves.	Multiple uses	*Open Space<br>*Ecological restoration (wildlife enclaves)<br>*Blue-green infrastructure	64,109
79.	United States	Chesapeake, Virginia	5	Coastal risks	Coastal restoration and migration space for natural shoreline features to migrate landward	*Coastal restoration	*Open Space<br>*^Ecosystem migration space*	110
80.	United States	Texas	4	Coastal risks	Public beach - Rolling easement. Coastal Erosion and Planning Act provides $50,000 for Texas property owners to realign houses from public beach areas to inland. Due to sea level rise, four houses originally inland of the high-water mark were found to trespass this mark and had to be removed.	*Open Space	Beach	44
81.	United States	Chilmark - Martha's Vineyard, Massachusetts	Infrastructure	Coastal risks	Coastal restoration - Beach renourishment following the removal of a revetment	*Coastal restoration	Beach renourishment following removal of revetment<br>*Open space	111
82.	United States	Austin, Texas	800	Erosion and water quality concerns, aiming to restore	The selection of flood risk reduction projects is guided by a Watershed Protection Master Plan which provides an assessment of Austin's erosion, flood, and water quality problems, prioritising solutions such as buyouts. Post-buyout land uses are determined by the city with the support of the community, including floodplain restoration, green infrastructure and open	Multiple uses – networked (strategic, integrated landscape planning)	*Open Space - Pollinator parks, community gardens<br>*Blue-green infrastructure<br>*Floodplain restoration<br>*^Habitat*	65,72,110

				natural floodplains and protect people and property.	space with neighbourhood gardens or planting of native grasses and wildflowers. Example of how conservation organizations can improve strategic retreat to successfully reduce risk and improve social and ecological resilience. [110]		*^Food production*	
83.	United States	Harris County, Texas	3,000 complete, 3,000 more planned	Riverine risks	Widen bayous, create recreational green spaces, and enhance stormwater drainage. Buyouts typically only occur if individual properties are a minimum of five acres, or if ten contiguous properties are acquired at once. Priority is given to areas suitable to create stormwater detention. This allows properties to be successfully converted to another use, reduces maintenance costs, and avoids checkerboarding within buyout areas. Equity concern: Difference in quality of open space in white and other neighbourhoods noted in research.	Multiple uses	*Open Space - Recreation<br>*Blue-green infrastructure (stormwater detention or floodplain restoration)<br>^Equity concerns	72,112,113
84.	United States	South Ocean Beach, San Francisco	Removing a highway and parking lot	Coastal risks	Removal of rock wall, road and parking lot, dune restoration, recreational enhancement (two lanes of highway converted to coastal trail) and protection of wastewater infrastructure.	Multiple uses	*Coastal restoration<br>*Open space - Recreation (new coastal trail)<br>*Protection measures - for wastewater infrastructure	104,114
85.	United States	San Antonio, Texas	400	Riverine risks	Open space. Difficulty getting some homeowners to participate in the program, partly due to mistrust among residents.<br><br>In the Wheatley Heights neighborhood the community redeveloped approximately 200 properties along the Salado Creek in 1998. A sports complex, including a football stadium, soccer fields, and cross-country trails, now occupies the area. "A plaque memorializes the people who contributed to the creation of the new sports complex while also remembering the flood event that destroyed the neighborhood. The place or the history of the neighborhood, as the only African American neighborhood in San Antonio (Pasley 2001), is missing from the memorial's narrative." (Zavar, 2019)	Multiple uses	*Open Space (sports complex, cross country trails)<br>*People remain in-situ<br>*Heritage preservation - plaque	2,59,73
86.	Austria	Linz, Danube floodplain	80	Riverine risks	Rezoning of the area from residential zones into grassland. Non-movers are restricted in the use of their buildings, such as no increase of actual and potential flood damage.	*Open Space	*People remain in-situ	2,115–117
87.	Austria	Danube river, Lower Austria	500	Riverine risks	Rezoning of the area from residential zones into grassland. Non-movers are restricted in the use of their buildings, such as no increase of actual and potential flood damage.	*Open Space	*People remain in-situ	2,116,118
88.	Germany	Moos, Bavaria	24	Riverine risks	Gradual demolition over time (a generational task). In some cases, agreements have been made that houses will be demolished when properties are inherited by the next generation. Demolition was responsibility of the homeowners, but the costs could be recouped from the state.	*Demolition	Phased/generational task	119
89.	Germany	Simbach at the Inn, Bavaria (Rottal-Inn)	11	Riverine risks	Improved structural protection and strategic open space planning - Community led redesign of the riverine area in the city centre as a green space and recreational area.	Multiple uses	*Protection measures<br>*Open Space – recreation and riverine areas	2,120
90.	Germany	Rostock, Mecklenburg-Vorpommern	540 ha	Coastal risks	Managed realignment, allowing the area to flood naturally following the removal of a pumping station. The coastline is expected to see an increase in habitat diversity, as well as a natural buffer zone of dunes and sand that will help reduce the impact of storms along the entire coastline. Conflicts between pre and post realignment use: the area was used as recreation space for walkers, cyclist, and bird watchers and as an entrance to the beach in front of the Hütelmoor. Public access to the Hütelmoor is now restricted, with access only via the forest. Wooden observation towers were installed at the forest edge to observe wildlife	Multiple uses	*Coastal restoration -dunes and saltmarsh<br>*Blue-green infrastructure (coastal buffer)<br>*Open Space (Nature reserve)<br>*^Reduced public access*	2,121
91.	Germany	Falshöft, Schleswig-Holstein	860 ha	Coastal risks	Lands rewetted behind an abandoned dike, and a smaller dike was built inland. Wild horse herds (Koniks) were introduced to maintain a semi-open landscape. 860 ha have been developed into a salt-water biotope as a	Multiple uses -	*Coastal restoration - saltmarsh	2,122,123

				compensation for nature losses. Water levels and discharges in the 860-ha large salt-water biotope are regulated by two tidal gates ~ 380 ha of the salt-water biotope are permanently water covered, 170 ha are intertidal flats, 215 ha salt marsh and about 95 ha by supratidal environments (artificially created bird islands). A decade after construction of the embankment, all characteristic Wadden Sea animal species and vegetation types and distributions reappeared in the salt-water biotope.		Managed realignment –<br>*Protection measures remain<br>*Blue-green infrastructure<br>*Open Space (Nature reserve)<br>^Offset - *Habitat restoration*	
92. Germany	Anklamer Stadtbruch, Olderhaff	1750 ha	Coastal risks	Managed realignment - Newly built landward dike	Multiple uses	*Coastal restoration – Lagoon<br>*Protection measure (remains)<br>*Blue-green infrastructure	124,125
93. Germany	Kreetsand, Elbe Estuary	30 ha	Coastal risks	Managed realignment - Breach dike and rely on former hinterland dike. Creation of recreational areas.	Multiple uses	*Coastal restoration- Mudflats<br>*Protection measure (remains)<br>*Open Space - Recreation<br>*Blue-green infrastructure	124,126
94. Germany	Sundische Wiese, Baltic Sea	940 ha	Coastal risks	Managed realignment - Breach dike and rely on former hinterland dike	Multiple uses	*Coastal restoration<br>*Protection measure (remains)<br>*Blue-green infrastructure	124
95. Germany	Polder Friedrichshagen, Bay of Greifswald	90 ha	Coastal risks	Managed realignment - Breach dike and rely on former hinterland dike	*Coastal restoration – Salt marsh	^Sea defence removal to reduce flood protection costs	124,125
96. Germany	Geltinger Birk, Baltic Sea	1000 ha	Coastal risks	Managed realignment - Breach dike and build new landward dike. A comprehensive tourism infrastructure was implemented, 30 km of walking paths, nature protection information and exhibition centre and kiosk, information stands and charts, guided tours inform about history, morphology and nature, wild Konik herd introduced. Traditional houses on the spit were transformed into seasonal holiday-flats.	Multiple uses	*Coastal restoration – Salt marsh<br>*Protection measure (remains)<br>*Blue-green infrastructure<br>*Open Space (walking paths, exhibition centre and kiosk, nature information stands, wild Konik herd<br>*Commercial: Holiday flats<br>^Tourism	2,122,124
97. Germany	Karrendorfer Wiesen, Baltic Sea	350 ha	Coastal risks	Managed realignment - Breach dike and build new landward dike	Multiple uses	*Coastal restoration – Salt marsh<br>*Protection measure (remains)<br>*Blue-green infrastructure	124
98. Scotland	Kennet Pans, Firth of Forth Estuary	8 ha	Coastal risks	Managed realignment - Breach dike and build new landward dike	Multiple uses	*Coastal restoration – Salt marsh<br>*Protection measure (remains)<br>*Blue-green infrastructure	124
99. Belgium	Lillo Potpolder, Scheldt Estuary	10 ha	Coastal risks	Managed realignment - Breach dike and build new landward dike	Multiple uses	*Coastal restoration – Salt marsh<br>*Protection measure (remains)<br>*Blue-green infrastructure	124
100. Belgium	Ketenisseschor, Scheldt Estuary	36 ha	Coastal risks	Managed realignment within summer polders	Multiple uses	*Coastal restoration – Salt marsh	124

							*Protection measure (remains)<br>*Blue-green infrastructure	
101.	Netherlands	Noarderleech / Noard Fryslân Bûtendyks, Wadden Sea	135 ha	Coastal risks	Managed realignment within summer polders	Multiple uses	*Coastal restoration – Salt marsh<br>*Protection measure (remains)<br>*Blue-green infrastructure	124
102.	Netherlands	De Noordwaard, North Brabant	75 ha	Riverine risks	Managed realignment - Depoldering of rural Noordward to make space for floodwaters. Retreat and adapt – some houses resettled in elevated mounds or outside of the area. Agricultural activities have reduced. New infrastructure such as bridges and pumping stations provide access and viewing points to connect the community with the new landscape. Recreation opportunities (cycling, boat visits and contemplation, plus the renovated Biesbosch Museum, a tourist attraction featuring the managed realignment landscape). New willow forest of two species of willow has been strategically planted to enable replacement of the concrete dyke with a lower one to reduce risks to the historic Steurgat fortress.	Multiple uses – networked (strategic, integrated landscape planning)	*Floodplain restoration<br>*Open space - Recreational<br>*Blue-green infrastructure<br>*Heritage preservation<br>*Redevelopment - (elevated houses)<br>*Protection and accommodation measures<br>^Tourism	12,127,128
103.	Portugal	Ria Formosa, Algarve	Not provided	Coastal risks	Lagoon system restoration and abandonment of salt farming. Creation of Ria Formosa Natural Park.	Multiple uses	*Coastal restoration – lagoon system<br>*Open Space	2,129
104.	England	Happisburgh, North Norfolk	9	Coastal risks	Clifftop enhancement project at Happisburgh (removal of beach debris, establish a buffer of open land, replace the car park, toilets, and beach access ramp) and realignment of the Cromer Footpath. Coastal Heritage project to help the local community know and record the threatened heritage, so they can interpret the village's historic, cultural, and environmental landscape. Properties on Beach Road (some blighted) were demolished once purchased. This allows for buildings to be demolished rather than waiting for them to be smashed on the beach below the eroding cliffs, supporting health and safety, natural beach character and orderly relocation.	Multiple uses	*Open Space<br>*Heritage preservation<br>^Blight reduction<br>^Health and safety, natural beach character and orderly relocation are promoted	58,130,131
105.	England	Trimingham Village Hall and Residences, North Norfolk	Unclear	Coastal risks	Demolition and some repurposing of heritage buildings - Dwellings no longer pose a threat of collapse onto the adjacent cliffs (a Site of Special Scientific Interest) with the risk of future beach debris and potential environmental hazards. Repurposing of the "Pilgrims Shelter" / Village Hall to be integrated into the Deep History Coast initiative, continuing to provide a local function.	Multiple uses	*Demolition<br>*Heritage preservation<br>*^ Repurposing*<br>*^Avoidance of effects on site of special scientific interest*<br>*^Health and safety, natural beach character and orderly redevelopment and relocation are promoted*	58,130
106.	England	East Riding, North Norfolk	12 households 43 structures demolished	Coastal risks	Demolition and site restoration of curtilage funded by project. Indirect benefits from reduction in potential for blight. Restoration of land following demolition of properties has restored land and prevented it from becoming wasteland - Foreshore environment protected from pollution, reducing impact on the natural environment and the requirement for compensatory habitat.	*Demolition and site restoration of curtilage	*^Reduction in potential for blight, pollution, and harm to ecosystems*	2,118,132
107.	England	Tollesbury, Essex	21-24 ha	Coastal risks	Managed realignment - Breach dike and build new landward dike. Salt-water marsh restoration. Due to fertilizers and other farming practices, species diversity may be lower in the reclaimed marshes.	Multiple uses	*Coastal restoration - Saltmarsh and mudflat restoration<br>*Blue-green infrastructure<br>*Protection measure (remains)<br>*^Habitat creation*	2,125,133,134

108.	England	Orplands, Essex	40 ha	Coastal risks	Managed realignment – Seawall breaches connecting the inter-tidal mudflat of the estuary with the burrow ditch.	Multiple uses	*Coastal restoration – Salt marsh<br>*Protection measure (remains)<br>*Blue-green infrastructure	135–137
109.	England	Abbotts Hall, Essex	87 ha	Coastal risks	Managed realignment - Breach dike and build new landward dike - creation of 87 hectares of saltmarsh, saline lagoons and grazing marsh resulting in a more sustainable coastal defence. New habitat created for water voles and sea slugs, and badgers, adders, grass snakes, lizards, and slow worms were moved under license. Certified archaeological excavations uncovered several 'red hills' (ancient salt workings), Roman pottery, a Dane encampment, and a Saxon bridge. Internationally important nature reserve for wildlife. Ramsar Site of Special Scientific Interest (SSSI), Special Areas of Conservation (SAC), Special Protection Areas (SPA).	Multiple uses	*Coastal restoration – Salt Marsh<br>*Open Space<br>*Protection measure (remains)<br>*Blue-green infrastructure<br>*Heritage preservation - Archaeological excavations<br>*^Habitat creation*<br>*^Species relocation.*	44,124,135,138–140
110.	England	Northey Island, Essex	0.8 ha	Coastal risks	Managed realignment - Breach and lowering<br>Nature reserve	Multiple uses	*Coastal restoration – Salt marsh<br>*Protection measure (remains)<br>*Blue-green infrastructure<br>*Open Space	135,141
111.	England	Thornham Point, West Sussex	6.75 ha	Coastal risks	Managed realignment - Initial accidental breach followed by management measures, relying on former hinterland dike	Multiple uses	*Coastal restoration – Salt marsh<br>*Protection measure (remains)<br>*Blue-green infrastructure	124,135
112.	England	Pawlett Hams, Somerset	4.8 ha	Coastal risks	Managed realignment - Breach dike and rely on former hinterland dike to reduce flood protection costs	Multiple uses	*Coastal restoration –Mud flat<br>*Protection measure (remains)<br>*Blue-green infrastructure	124,135
113.	England	Steart Marsh, Somerset	250 ha	Coastal risks	Managed realignment -Excavation of a creek network and pools, the construction of new flood defence embankments and raising of a small length of existing embankment. The Wildfowl & Wetland Trust now manages the site as a nature reserve and farmland. A reduction in intense grazing and farming practices has resulted in more diverse vegetation structure. A mixed grazing regime using local graziers, to include longhorn cattle, Dexter cattle, Friesian cattle, and low numbers of Rutland sheep. Visiting facilities and walkway access provides value as an educational facility. The finished project enables flood risk reduction, intertidal habitat for wildlife and a space for public use. Development across the Severn Estuary has caused considerable habitat loss and coastal squeeze. National and European directives require the Environment Agency to offset intertidal habitat losses with compensatory habitat. This site can compensate for 40% of the total habitat losses in the region. Steart provides accommodation space for sediment retention and habitat transition as an ecosystem service.[142]	Multiple uses	*Coastal restoration - Lagoon, mudflat, saltmarsh<br>*Open Space (Nature Reserve)<br>*Farming continues (drystock)<br>*Blue-green infrastructure<br>*Protection measure (remains)<br>*^Offset - habitat creation to offset intertidal habitat loss)*<br>*^Ecosystem migration space*<br>*^Carbon accumulation*	124,135,142–145
114.	England	Bleadon Levels, Somerset	13 ha	Coastal risks	Managed realignment - Wessex Water created the saltmarsh at Bleadon Levels in 2000 on land previously used for growing maize, by breaching the Victorian sea defence and excavating a network of tidal creeks. A bigger sea defence was created inland, with the area between that and the River Axe being developed into the saltmarsh habitat. Wessex Water is looking to introduce cattle to the area to help manage the site.	Multiple uses	*Coastal restoration– Salt marsh<br>Breach and lowering of defences - *Protection measure (remains)<br>*Blue-green infrastructure<br>*Open Space – Nature reserve with potential use of cattle for land maintenance	124,135,146

115.	England	Lantern Marsh, Ore Estuary, Suffolk	29-37 ha	Coastal risks	Managed realignment - Breach	Multiple uses	*Coastal restoration – Salt marsh<br>*Protection measure (remains)<br>*Blue-green infrastructure<br>*Open Space - Estuary	124,135
116.	England	Watertown Farm, Devon	1.5 ha	Coastal risks	Managed realignment - Breach	Multiple uses	*Coastal restoration – Salt marsh<br>*Protection measure (remains)<br>*Blue-green infrastructure	135
117.	England	Annery Kiln, Devon	3.8 ha	Coastal risks	Managed realignment - Breach	Multiple uses	*Coastal restoration – Salt marsh<br>*Protection measure (remains)<br>*Blue-green infrastructure	135
118.	England	Goosemoor, Exe Estuary, Devon	6 ha	Coastal risks	Managed realignment – no specific detail on breach/removal of structures	*Coastal restoration– Salt marsh	*Blue-green infrastructure	135
119.	England	Pillmouth, Suffolk	12.9 ha	Coastal risks	Managed realignment - Breaches	Multiple uses	*Coastal restoration – Salt marsh<br>*Protection measure (remains)<br>*Blue-green infrastructure	135
120.	England	Brancaster West Marsh, North Norfolk	8 ha	Coastal risks	Managed realignment - Partial bank - initial accidental breach followed by management measures, gabion removal and dune reprofiling. Residents and right holders concerned about potential loss of footpaths caused by the establishment of a creek following the managed realignment works.	Multiple uses	*Coastal restoration– Salt marsh<br>*Open Space (beach access)<br>Breach of defences<br>*Protection measure (remains)<br>*Blue-green infrastructure<br>^ Concerns about reduced access	124,125,147
121.	England	Freiston Shore, Lincolnshire	70 ha	Coastal risks	Managed realignment – strengthen part of the secondary line of defence and to construct a realigned bank on the remainder, creating more than 70 ha of saltmarsh, including mudflats and a lagoon for habitats and flood defence purposes. The area is a Site of Special Scientific Interest (SSSI), Special Protection Area (SPA) and Ramsar site. The lagoon attracted around 25,000 visitors in its first year, and with the erection of interpretation boards is providing educational benefits. The project also provided access improvements for bicycles and the disabled, car park facilities and general conservation reserve improvements (8 km of trails, hides and viewpoints, education centre).	Multiple uses	*Coastal restoration – Salt marsh<br>*Open Space (car + bicycle parks, education centre, trails, accessibility improvements, viewpoints)<br>*Protection measure (remains)<br>*Blue-green infrastructure	135,148,149
122.	England	Roach Estuary, Jubilee Marsh, Allfleet's Marsh, Dynamic Lagoons (Wallasea Island)	800 ha	Coastal risks	Comprehensive, integrated managed realignment planning, land raising and coastal restoration for flood risk reduction and to compensate for historic losses of habitat in the region. Multiple co-benefits: tourism, open space, international habitat protection, offset, carbon accumulation and landfill diversion via waste management partnership for infrastructure earthworks. Provides accommodation space for sediment retention and habitat transition as an ecosystem service - with long shallow slopes allowing for the saltmarsh to creep up as sea levels rise. Grazing animals on site provide an income	Multiple uses – Networked (strategic, integrated landscape planning)	*Coastal restoration – salt marsh<br>*Blue-green infrastructure<br>*Protection measures<br>*Open Space<br>Farming – grazing animals<br>^Carbon accumulation<br>^Repurposing - Landfill material diversion	124,135,142,150–152

				stream to the farmer. The project offers guided walks and nature trails, and this increase in visitors has benefitted local shops.		^Offset - Compensatory habitat creation due to development<br>^Ecosystem migration space<br>^Economic benefits from tourism and farming	
123.	England	Jaywich, Tendring	4	Coastal risks	Four properties purchased and demolished at Jaywick. Considered buy and lease back but no interested Registered Social Landlords.	*Demolition	^Reducing future blight	118,132
124.	England	Waveney	1	Coastal risks	Demolition, minor positive impact in terms of reducing future blight	*Demolition	^Reducing future blight	118,132
125.	England	Severn Estuary, Cone Pill	50 ha	Coastal risks	Managed realignment - Newly built landward dike. Agriculture continues, site semi-returning to wetland.	Multiple uses	*Coastal restoration<br>*Farming continues<br>*Protection measure (remains)<br>*Blue-green infrastructure	124,153
126.	England	Paull Holme Strays, Humberside	80 ha	Coastal risks	Managed realignment - Newly built landward dike creating 80 ha of intertidal area, increasing sediment storage capacity. Nature reserve with trails. Limited wheelchair accessibility.	Multiple uses	*Coastal restoration – Salt marsh, mudflat, and transitional grassland restoration<br>*Protection measure (remains)<br>*Blue-green infrastructure<br>*Open Space	2,124,135,154,155
127.	England	Easton Broad, North Sea	130 ha	Coastal risks	Managed realignment - Newly built landward dike	Multiple uses	*Coastal restoration<br>*Protection measure (remains)<br>*Blue-green infrastructure	124
128.	England	Tees Estuary, Greatham <u>North</u>	40 ha	Coastal risks	Managed realignment - Newly built landward dike	Multiple uses	*Coastal restoration – Salt marsh and mudflat<br>*Protection measure (remains)<br>*Blue-green infrastructure	124
129.	England	Tees Estuary, Greatham <u>South</u>	40 ha	Coastal risks	Managed realignment - Newly built landward dike	Multiple uses	*Coastal restoration – Salt marsh and mudflat<br>*Protection measure (remains)<br>*Blue-green infrastructure	124
130.	England	Titchwell Marsh, North Sea	11 ha	Coastal risks	Managed realignment - Breach dike and rely on former hinterland dike. Nature reserve with visitor centre, café, nature trails, viewpoints, wheelchair and family accessible toilets and trails, and education centre.	Multiple uses	*Coastal restoration – Salt marsh<br>*Protection measure (remains)<br>*Blue-green infrastructure<br>*Open Space (café, trails, accessibility features, education centre)	124,156
131.	England	Alnmouth Estuary	28 ha	Coastal risks	Managed realignment - Breach dike and rely on former hinterland dike	Multiple uses	*Coastal restoration – Salt marsh<br>*Protection measure (remains)<br>*Blue-green infrastructure<br>*Open Space Estuary	124
132.	England	Littlehaven Beach, South Tyneside, Northeast England	Seawall	Coastal risks	Managed realignment - seawall realignment changed the shoreline from a protruding to a concave planform, reducing maintenance costs and increasing amenity value	Multiple uses	*Coastal restoration<br>*Protection measure (remains)	126

						*Open space<br>^Improved amenity value	
133. England	Brownsea Island, Dorset	Removal of coastal protection structures	Coastal risks	Managed realignment -removal of coastal protection structures and renaturalising coastline for recreation, habitat, and safety.	*Coastal restoration	*Open space<br>*^Habitat*<br>*^Health and safety*	126
134. England	Dyfi Estuary, Yns-hir	6 ha	Coastal risks	Managed realignment - Breach dike and rely on former hinterland dike	Multiple uses	*Coastal restoration – Salt marsh<br>*Protection measure (remains)<br>*Blue-green infrastructure	124,157
135. England	Hesketh Outmarsh West: Ribble estuary, Northwest England	180 ha	Coastal risks	Managed realignment - Breach dike and rely on former hinterland dike. Historic creek beds were dredged, lagoons were dug, and the inner wall was reinforced. Increased recreation opportunities. The new saltmarsh reduces the flood risk to 143 properties and nearby farmland. Nature reserve includes trails, viewpoint, parking and pram and wheelchair access. Carbon accumulation in accreting sediments.	Multiple uses -	*Coastal restoration – Saltmarsh<br>*Open Space (Nature Reserve)<br>*Blue-green infrastructure<br>*Protection measure (remains)<br>^Carbon accumulation	2,124,158,159
136. England	Hesketh Outmarsh East: Ribble estuary, Northwest England	160 ha	Coastal risks	Managed realignment - Breach dike and improve hinterland dike. The 'Hesketh Out Marsh East' realignment project was created through seawall breaching in September 2017. Additional work has involved strengthening and raising the height of 2km of flood banks, reducing the flood risk to more than 140 properties and 300 hectares of farmland nearby. This project is a partnership between the RSPB, Natural England and the Environment Agency producing a new coastal reserve that is managed by the RSPB. Nature reserve includes trails, viewpoint, parking and pram and wheelchair access. Carbon accumulation in accreting sediments.	Multiple uses	*Coastal restoration - Saltmarsh and lagoons<br>*Open Space (coastal reserve, trails, viewpoint and wheelchair and family accessibility)<br>*Blue-green infrastructure<br>*Protection measure (remains)<br>^Carbon accumulation	2,124,158–160
137. England	Medmerry: Bracklesham, West Sussex	302 ha (183 ha new habitat)	Coastal risks	Managed realignment - Dike realigned landward and new wetland/nature reserve created. As well as reducing flood risk in the area, the new habitat of mudflat, saltmarsh, and transitional grassland offsets losses of similar habitats across the shoreline of the northern Solent. Land was acquired by the Environment Agency and is now managed by the Royal Society for the Protection of Birds as a reserve for biodiversity interests. A 10km public footpath runs along the top of the new flood bank, with four viewpoints, plus a carpark. Recent larger-scale MR initiatives in England, such as at Medmerry, provide accommodation space for sediment retention and habitat transition as an ecosystem service.[142]. Limited geological conservation funding or regulations	Multiple uses	*Coastal restoration - Saltmarsh, mudflat, transitional grassland<br>*Open Space (Nature reserve)<br>*Blue-green infrastructure<br>*Protection measure (remains)<br>^Offset - Habitat creation<br>^ Flood risk reduction<br>^ Ecosystem migration space<br>^Limited geoconservation (Heritage)	2,124,142,161–163
138. England	Belle Tout lighthouse, Beachy Head, East Sussex	1	Coastal risks	Eroding cliff edge – In 1999 the lighthouse (which had been converted to a private home) was moved 17 m away from the edge of the eroding cliff, privately funded.	*Residential Setback		126
139. France	Sète to Marseillan Road, Occitania	Road	Coastal risks	Dune stabilisation and restoration planting - dunes were made more stable using bundles of brushwood, and they now protect the lido. Over 310,000 marram grass were planted. Managed retreat was supported by demand for income from tourism and the support for the well-being of inhabitants, linked to the stability of the beach, and because houses did not require relocating.	*Coastal restoration	*Open space<br>^Tourism and social well-being benefits of coastal restoration	164,165
140. France	Criel-sur-Mer, Normandy	14	Coastal risks	Demolition of buildings	*Demolition		164

141.	France	Wimereux, Pas-de-Calais	5	Coastal risks	Demolition of buildings	*Demolition		164
142.	Solomon Islands	Nusa Hope, New Georgia	35 (261 people)	Coastal risks	Partial relocation. Relocating families re-used building materials from their existing homes, supplementing this with natural building materials (sago leaf, hardwood and palm trunks) from the new settlement areas.	*People remain in-situ	^Repurposing	2,166
143.	Solomon Islands	Nuatambu, Choiseul Province	24 (133 people)	Coastal risks	71% of population relocated between 2007-2016. Community members that have remained on Nuatambu expressed their need to remain as long as possible on the island, to act as guardians and preserve cultural relations.	*People remain in-situ		166
144.	Fiji	Vunidogoloa, Koroalau	26	Coastal risks	Abandonment – The original village of Vunidogoloa is abandoned. Houses, now dilapidated, are overgrown with vegetation. The villagers still regularly visit the old village.	*Abandonment but continued access to site (relational co-evolution)		2,167–171
145.	Fiji	Narikoso, Ono Island	7	Coastal risks	Area is wet at full tide. Wider area impacted by reef and mangrove destruction and erosion from the development of the new site.	*Sea Change	^Environmental destruction from retreat	2,169,171,172
146.	Fiji	Tabuya, Kadavu	2 (ongoing)	Coastal risks	Transition phase - Some relocation with continued access to old site once complete. Continued access to the original village is considered as an integral part of the community resettlement.	*People remain in-situ (relational co-evolution)		173
147.	Fiji	Denimanu Village, Yadua Island	19	Coastal risks and landslide Cyclone	Two rows of houses were destroyed in a few hours during Tropical Cyclone Evan in 2012. The eroding shoreline is only a few metres from the edge of another row of houses and the sea often floods part of the village during high spring tides. Most participants stated they would eventually relocate their homes further inland and would consider planting mangroves along the shore front to absorb wave energy. Landslide occurred behind Denimanu in March 2017 necessitating the abandonment of the island's primary school.	*Sea Change (potential mangrove planting in future)<br>*Abandonment - school		169,171,174
148.	Australia	Grantham, Queensland	115	Riverine/Flash flooding	Removal of all buildings (by property owners who participated in the land swap. Limited development zone - contains some houses where there are existing development entitlements, but primarily provides a range of low-key rural activities which are agricultural in nature and which are compatible with the remaining residential uses (e.g. flower farms, plant nurseries, turf farming, garden supplies, equine uses)	*Open Space (rural activities e.g. agriculture, flower farming, garden suppliers, equine uses, park)	*People remain in-situ	2,175,176
149.	Aotearoa New Zealand	Project Twin Streams: Waitakere, Auckland	78 relocations and 78 partial property purchases.	Riverine risks and holistic project goals	Guided by the holistic 'Eco City' mandate, strategic, integrated planning was undertaken to manage water supply, wastewater and stormwater, stream, and harbour pollution, restore ecological corridors, mitigate climate change, provide pedestrian and cycle links and foster sharing of diverse cultures through storytelling, art, music, literature and drama. Strategic acquisitions enabled floodplain restoration, BGI, riparian planting and covenants, esplanade reserves, drainage reserves, public parks, walkways and community orchards and gardens. Other initiatives include the art trail, the first community based Pā Harakeke in the city, enabling cultural flax harvest and weaving education, Te Kawerau a Maki Identity Marker - pou ihi representing the relationship between water and people, Te Māra Rongoā (Medicinal Garden). Temporary leasebacks.	Multiple uses – Networked (strategic, integrated, landscape planning)	*Floodplain restoration (Riparian planting and covenants -protected area)<br>*Blue-green infrastructure (Esplanade and drainage reserves)<br>*Open Space (Public parks, trails, community orchards and gardens utilising existing fruit trees, cultural gardens, art and environmental education)<br>*Residential Leasebacks<br>*Heritage preservation – Cultural landscapes - Pā harakeke, Te Māra Rongoā and pou ihi - weaving education and garden, Māori medicinal gardens, environmental education, Māori worldview.<br>*^Food production*	2,177–183

						^*Habitat creation*	
150. Aotearoa New Zealand	Franz Josef/Waiau, West Coast	Lodge and campground	Riverine risks	Road and river management - Restrictions were placed on the titles of the land to prohibit future residential or commercial accommodation. A Memorandum of Understanding was agreed to specify that the land on the south side be for road and river management purposes only	*Open Space		178,179
151. Aotearoa New Zealand	Muriwai Beach, Auckland	Carpark and surf club	Coastal risks	Asset removal (carpark and surf club) and coastal restoration (dune planting)	*Coastal restoration	*Open space - beach	178,179
152. Aotearoa New Zealand	Tauranga, Bay of Plenty	1	Riverine risks	Constrained overflow path or ponding area	*Open Space	*Blue-green infrastructure	178,179
153. Aotearoa New Zealand	Okariha/Sunset Beach, Waikato	Community hall and lifesaving tower	Coastal risks	Dune reconstruction, planting and new access ways. Removal of debris from the beach.	*Coastal restoration	*Open space	178,179,184–186
154. Aotearoa New Zealand	Edgecumbe, Bay of Plenty	12	Riverine risks	Property purchase to realign a stop bank. Residential structures removed and land restored to grass field. "Contractors retrieved a section of the old stop bank featuring an eel mural. This, and a section of stop bank discovered in the shape of a heart, were both placed in the reserve area as a memorial… When the footpaths were constructed through the reserve section, the numbers of the houses which had been permanently removed were stamped in the kerbs as a remembrance of where they had been. This was done by the previous house owners and was a very moving occasion. The land was then purchased by the BOPRC to make this a reserve area as a memorial for future generations to remember what had happened that day."[187]	Multiple uses	*Open space<br>*Protection measure - realigned stop bank and reserve<br>*Heritage preservation: memorial – stop bank mural and curb stamps)	178,179,187,188
155. Aotearoa New Zealand	Queen Elizabeth Park, Wellington	Public infrastructure and amenities	Coastal risks	Coastal restoration - Removal of physical assets (surf club, parking and picnic areas) closing of some walking tracks and restoration of foredunes.	*Coastal restoration	*Open space	178,179,189,190
156. Aotearoa New Zealand	Christchurch, Canterbury	8,000	Geological - Earthquake, liquefaction and cliff instability and rock fall	<u>Ōtākaro Avon River Corridor Specific Purpose Zone:</u> recreation, cultural and community-based activities; visitor and small-scale retail activities; limited residential development on the outer edge of the Zone to improve integration between existing neighbourhoods; varied learning, experimenting and research opportunities, including testing and demonstrating adaptation to natural hazards and climate change; transitional activities and structures; the continuation of pre-earthquake activities on privately-owned properties that still exist.<br><br>*Networked spatial planning - Ōtākaro Regeneration Plan and Greenprint spatial plan with a combination of land uses, projects and activities including: 350 hectares of native ecological restoration for native birds and mahinga kai, 200,000 trees to be planted, 80 hectares of wetlands to treat 2,600 hectares of stormwater catchment to improve water quality, stop banks to reduce risk from flooding for up to 4,000 homes, with design flexibility to adjust to sea level rise, ~150 sections for 'edge housing' to reconnect the Regeneration Area and local neighbourhoods, six areas to trial adaptable housing. • An east–west road and bridge, which would improve community connectivity and resilience. • An eleven-kilometre walking/cycle City to Sea path connecting communities with the city, river flatwater sports. footbridges to reconnect communities across the river, a Cultural Trail expressing identity and history, eight landings that provide connections to the river and that,*	Multiple uses – networked (Ōtākaro and Waimakariri – (strategic, integrated landscape planning)	*Floodplain restoration (tree planting, wetlands, ecological restoration)<br>*Open Space (Community gardens/orchards, foraging zone, fungi farm, river sports, eco-hub, playground, berry farms, propagating apricot trees, cropping, grazing, keeping beehives and chickens, and nursing plants, nature zones, memorial garden, BMX track, dog park, coastal park<br>*Commercial - social enterprise, commercial and retail activities, home business, car parking and/or a motor caravan park, community and business leases<br>*People remain in-situ<br>*Redevelopment – edge development, adaptable	179,191–199

				depending on their location, may include bike and kayak hire, toilets, carparks, cafes and retail. Current activities: Avebury House - social enterprise fostering community connections and provides educational, social and cultural activities, community garden, eco-hub, fungi farm, forest park, Sutton Heritage House and Garden, Adventure Ave playground, edible foraging.<br><br>Southshore and Brooklands: Specific Purpose Zone: Existing residential and commercial activities, home businesses, market/community gardens, existing and new community activities/facilities, temporary activities and hazard management (Southshore South New Brighton – plans for a new bund, erosion protection structures and a cobble beach on the estuary edge to reduce flood and erosion risks.)<br><br>Port Hills- Mixture of open space zones (natural and community parks), vacant rural and residential port hills zoned land (some of which may be sold privately, or social housing properties redeveloped) and some land is leased for community purposes (berry farms, propagating apricot trees, sheep grazing, keeping beehives and chickens, and nursing plants)<br><br>Waimakariri - Open Space – 41 hectares, with various uses including sports fields, parks, a heritage and mahinga kai area, managing the land as part of the existing Tūhaitara Coastal Park, a memorial garden for ash internment, BMX track and/or dog park; Private lease – 2 hectares of open space/non-permanent/flood tolerant activities, potentially including boat storage, tennis courts and/or car parking. Business – 9 hectares, including commercial and retail activities, car parking and/or a motor caravan park; Rural – 30 hectares, likely to include cropping and grazing, but excluding intensive farming; Infrastructure – Dudley drain		housing, port hills social housing and individual sites Infrastructure – bridges and drains<br>*Protection measures – stop banks, seawalls/bunds<br>*Blue-green infrastructure (wetlands)<br>*Heritage preservation – cultural landscapes - mahinga kai, cultural trails, heritage house and garden<br>*Demolition - vacant land<br>*^Food production*<br>*^Habitat*<br>*^Cultural identity and heritage*<br>*^Economic (housing, commerce, retail, leases)*<br>*^Education and research (adaptable housing, climate change adaptation), fungi farm*<br>*^Water quality BGI*<br>*^Risk reduction*	
157.	Aotearoa New Zealand	Flockton Basin, Canterbury	6	Riverine risks	Retreat, mitigate and redevelop - Structural demolition with some ability for redevelopment following flood pump and drainage improvements.	*Redevelopment	Following risk reduction measures	179,200
158.	Aotearoa New Zealand	Matatā, Bay of Plenty	34 properties (16 homes)	Geological - Debris flow	Open Space Coastal Protection Zone with controls on land use activities due to debris flow risk. Danger signs to advise of debris flow risk. All activities e.g. residential, commercial, community) are prohibited – only transitory recreational use of open space or specifically identified low risk activities are allowed. There is a risk to life for visitors to the area.	*Open Space (transitory recreational only – risk to life)		179,201,202
159.	Aotearoa New Zealand	Riverlink: Hutt City, Wellington	75	Riverine risks	Integrated spatial and strategic planning encompassing managed realignment and urban revitalisation, to make room for the river, address limits to structural protection, enable greater flood water attenuation and provide amenity and urban revitalisation benefits. Key design elements include wetlands at stormwater outlets along the river margin to attenuate and treat urban stormwater, enhancement of biological diversity and habitat by restoring native vegetation in the floodplain, improving connections to the river with pathways from the city centre, and urban spaces that people can actively and passively engage with the river (e.g. water sports, seating/terraces, river stepping stones) encompassing historic and cultural values in the design to enhance cultural expression of Māori and later settlement patterns and stories. RiverLink transitions the existing flood corridor into a linear park providing diverse amenities and uses. Enhancing ecology in the urban centre benefits the ecology of the entire city, enhances the connection to the region and contributes to a cohesive urban identity.	Multiple uses – networked (strategic, integrated landscape planning)	Managed realignment –<br>*Protection measure - stop bank<br>*Redevelopment (Pedestrian and cycling bridge, urban esplanades)<br>*Blue-green infrastructure (wetlands)<br>*Floodplain restoration (River restoration planting and wetlands)<br>*Open Space (linear park)<br>*Heritage preservation (cultural and historic design principles)	179,203–205

160.	Aotearoa New Zealand	Waihi Beach, Bay of Plenty	'Several houses'	Coastal risks	Set back - houses relocated within sites	*Residential Setback		206
161.	Papua New Guinea	Bouganville, Carteret Islands	8 (to date)	Coastal risks	Some relocation and plans for a Marine Protected Area to preserve and protect cultural biodiversity and Indigenous practices.	Multiple uses planned (Networked seascape scale)	*Coastal restoration/ conservation (Marine Protected Area Plan – relational co-evolution) *People remain in-situ	12,207,208

Notes
* = Functional code
^ = Broad positive and adverse outcomes evident that are not directly captured by the functional typologies
Functions are noted as evidenced in the academic and grey literature, therefore there may be additional functions not captured by this review.

Table S2: Methods overview

'Scopus' search using the following terms	Screening	Results
"Managed retreat" n = 233	Records unable to be accessed n = 7<br>Duplicates removed n = 3<br>Total primary records reviewed n = 263	Records reviewed and excluded n = 168<br>168 papers examined planned retreats but they either did not include case studies, or insufficient detail was provided on the case location or approach, or PR was planned but not initiated, or PR was implemented for reasons other than reducing risks to people, or it entailed climate migration (movement across borders).<br>Selected records n = 98<br>Cases studies were found in 98 articles. In these cases, secondary sources (sources cited in the primary record) were also investigated to secure supplementary cases and information. In addition, internet searches were undertaken in search of project webpages and reports if further detail was required to clarify the land use category or planning mechanisms applied.
"Planned retreat" n= 40		
Total results n = 273	Total results after screening n = 263	Selected records = 98

Table S3: Regional land use types and risks

Number of case studies	Country	Risk type	Land/sea use types identified post-retreat
Africa			
1	Malawi	Riverine	*People remain in-situ ,*Farming continues
1	Mozambique	Riverine	*People remain in-situ, *Farming continues
1	Zambia	Riverine	*People remain in-situ
Asia			
3	China	Multi-risk	*Farming continues, *Floodplain and ecological restoration
2	India	Coastal, riverine	*Redevelopment, *BGI, *People remain in-situ
2	Indonesia	Coastal, riverine	*People remain in-situ, *Reoccupation
1	Iran	Geological- earthquake	*People remain in-situ, *Farming continues
1	Japan	Coastal (tsunami)	*Protection and accommodation measures, *Coastal restoration, *Open Space, *Heritage preservation<br>*Farming continues
6	Philippines	Coastal, riverine, Typhoon	*Reoccupation, *Open Space
1	Malaysia	Coastal	*Sea Change
1	Nepal	Geological-Landslide	*Abandonment
1	Sri Lanka	Coastal	*People remain in-situ, *Redevelopment, *Protection measures (+ remaining), *Coastal restoration, *Open Space
1	Taiwan	Geological-Landslide	*Heritage preservation
1	Vietnam	Coastal	*Sea Change
Europe			

2	Austria	Riverine	*Open Space<br>*People remain in-situ
2	Belgium	Coastal	*Coastal restoration, *Protection measure (remains), *BGI
35	England	Coastal	*Open Space, *Heritage preservation, *Demolition, *Coastal restoration, *BGI, *Protection measure (remains), *Farming continues, *Residential setback
3	France	Coastal	*Open Space, *Coastal restoration, *Demolition
10	Germany	Coastal, riverine	*Demolition<br>*Protection measures<br>*Open Space<br>*BGI<br>*Coastal restoration<br>*Redevelopment
2	Netherlands	Coastal, riverine	*Coastal restoration<br>*Protection measure (remains)<br>*BGI<br>*Floodplain restoration<br>*Open Space<br>*Heritage preservation<br>*Redevelopment<br>*Protection and accommodation measures
1	Portugal	Coastal	*Coastal restoration<br>*Open Space
1	Scotland	Coastal	*Coastal restoration<br>*Protection measure (remains)<br>*BGI
North America			
6	Canada	Coastal, riverine	*Open Space<br>*BGI<br>*Coastal restoration<br>*Protection measure (remains)<br>*Heritage preservation
1	Guatemala	Geological- Debris flow	*Open Space
47	USA	Coastal, riverine, geological-landslide, hurricane, erosion and water quality	*Open Space<br>*People remain in-situ<br>*Coastal restoration<br>*Ecological restoration<br>*Redevelopment<br>*Reoccupation<br>*Sea Change/transition phase<br>*Heritage preservation<br>*Protection measures<br>*BGI<br>*Floodplain (and creek) restoration and reconnection<br>*Farming continues<br>*Residential leasebacks<br>*Residential amalgamations<br>*Abandonment

			*Commercial<br>*Demolition
South America			
1	Argentina	Riverine	*Open Space
3	Brazil	Coastal, riverine	*Sea Change<br>*Open Space<br>*Redevelopment<br>*Coastal restoration
3	Colombia	Coastal, riverine, geological-land instability, erosion, and discharge issues	*Ecological restoration<br>*Open Space<br>*Sea Change
1	Peru	Riverine	*Commercial
Oceania			
12	Aotearoa New Zealand	Coastal, riverine, geological - debris flow, earthquake, liquefaction, cliff instability and rock fall	*Open Space<br>*Floodplain restoration<br>*Coastal restoration<br>*BGI<br>*Residential leasebacks<br>*Heritage preservation<br>*Protection measures<br>*Commercial<br>*People remain in-situ<br>*Redevelopment<br>*Demolition
1	Australia	Riverine	*Open Space<br>*People remain in-situ
4	Fiji	Coastal, geological-landslide, Cyclone	*Abandonment (with some continued access)<br>*People remain in-situ<br>*Sea Change
1	Papua New Guinea	Coastal	*Coastal restoration<br>*People remain in-situ
2	Solomon Islands	Coastal	*People remain in-situ

Table S1 References

1. Funder, M., Mweemba, C. & Nyambe, I. The Politics of Climate Change Adaptation in Development: Authority, Resource Control and State Intervention in Rural Zambia. *J. Dev. Stud.* 54, 30–46 (2018).
2. Ajibade, I., Sullivan, M., Lower, C., Yarina, L. & Reilly, A. Are managed retreat programs successful and just? A global mapping of success typologies, justice dimensions, and trade-offs. *Glob. Environ. Change* 76, 102576 (2022).
3. Artur, L. & Hilhorst, D. Floods, resettlement and land access and use in the lower Zambezi, Mozambique. *Land Use Policy* 36, 361–368 (2014).
4. Nicholson, H. Resistance, acceptance, and misalignment of goals in climate-related resettlement in Malawi. in *Global Views on Climate Relocation and Social Justice* (Routledge, 2021).
5. Lei, Y., Finlayson, C. M., Thwaites, R., Shi, G. & Cui, L. Using Government Resettlement Projects as a Sustainable Adaptation Strategy for Climate Change. *Sustainability* 9, (2017).
6. Pittock, J. & Xu, M. *Controlling Yangtze River Floods: A New Approach. World Resources Report Case Study.* (2011).
7. Göransson, G., Van Well, L., Bendz, D., Danielsson, P. & Hedfors, J. Territorial governance of managed retreat in Sweden: addressing challenges. *J. Environ. Stud. Sci.* 11, 376–391 (2021).
8. Du, F. Ecological Resettlement of Tibetan Herders in the Sanjiangyuan: A Case Study in Madoi County of Qinghai. *Nomadic Peoples* 16, 116–133 (2012).
9. Kumar, N. Incentives and expectations: community resiliency and recovery in Tamil Nadu after the Indian Ocean tsunami. *Indep. Rev.* 22, 135–152 (2017).
10. Ahmed, I. & McEvoy, D. Post-tsunami resettlement in Sri Lanka and India: Site planning, infrastructure and services. *International Journal of Disaster Resilience in the Built Environment,* 5, 53–65 (2014).
11. Andrew, S. A., Arlikatti, S., Long, L. C. & Kendra, J. M. The effect of housing assistance arrangements on household recovery: an empirical test of donor-assisted and owner-driven approaches. *J. Hous. Built Environ.* 28, 17–34 (2013).
12. Hino, M., Field, C. B. & Mach, K. J. Managed retreat as a response to natural hazard risk. *Nat. Clim. Change* 7, 364–370 (2017).
13. Cronin, V. & Guthrie, P. Community-led resettlement: From a flood affected slum to a new society in Pune, India. *Environ. Hazards* 10, (2011).
14. Badri, S. A., Asgary, A., Eftekhari, A. r. & Levy, J. Post-disaster resettlement, development and change: a case study of the 1990 Manjil earthquake in Iran. *Disasters* 30, 451–468 (2006).
15. Taylor, J. A tale of two cities: comparing alternative approaches to reducing the vulnerability of riverbank communities in two Indonesian cities. *Environ. Urban.* 27, 621–636 (2015).
16. Tadgell, A., Doberstein, B. & Mortsch, L. Principles for climate-related resettlement of informal settlements in less developed nations: a review of resettlement literature and institutional guidelines. *Clim. Dev.* 10, 102–115 (2018).
17. Birkmann, J. *et al.* Extreme events and disasters: a window of opportunity for change? Analysis of organizational, institutional and political changes, formal and informal responses after mega-disasters. *Nat. Hazards* 55, 637–655 (2010).
18. Matsumaru, R., Nagami, K. & Takeya, K. Reconstruction of the Aceh Region following the 2004 Indian Ocean tsunami disaster: A transportation perspective. *IATSS Res.* 36, 11–19 (2012).
19. Carrasco, S., Ochiai, C. & Okazaki, K. Disaster Induced Resettlement: Multi-stakeholder Interactions and Decision Making Following Tropical Storm Washi in Cagayan de Oro, Philippines. *Int. Inst. Infrastruct. Renew. Reconstr. I3R2* 218, 35–49 (2016).

20. Santiago, J. S. S., Manuela, W. S., Tan, M. L. L., Saňez, S. K. B. & Tong, A. Z. U. Agency-driven post-disaster recovery: A comparative study of three Typhoon Washi resettlement communities in the Philippines. *Int. J. Disaster Risk Reduct.* 27, 480–489 (2018).

21. Iuchi, Kanako & Maly, Elizabeth. Residential Relocation Processes in Coastal Areas: Tacloban City after Typhoon Yolanda. in *Coming Home after Disaster* (Routledge, 2016).

22. See, J. & Wilmsen, B. Just adaptation? Generating new vulnerabilities and shaping adaptive capacities through the politics of climate-related resettlement in a Philippine coastal city. *Glob. Environ. Change* 65, 102188 (2020).

23. Siders, A. R., Ajibade, I. & Casagrande, D. Transformative potential of managed retreat as climate adaptation. *Curr. Opin. Environ. Sustain.* 50, 272–280 (2021).

24. Thomas, A. *Resettlement in the Wake of Typhoon Haiyan in the Philippines: A Strategy to Mitigate Risk or a Risky Strategy?* https://www.brookings.edu/wp-content/uploads/2016/06/Brookings-Planned-Relocations-Case-StudyAlice-Thomas-Philippines-case-study-June-2015.pdf (2015).

25. Boano, C. Housing anxiety and multiple geographies in post-tsunami Sri Lanka. *Disasters* 33, 762–785 (2009).

26. Samaranayake, R. a. D. B. Pre-and post-tsunami coastal planning and land-use policies and issues in Sri Lanka. in *RAP Publication (FAO)* (FAO, 2007).

27. United Nations Environment Programme. *UNEP Post-Tsunami Recovery Activities 2004-2007*. https://reliefweb.int/report/indonesia/unep-post-tsunami-recovery-activities-2004-2007 (2008).

28. Centre for Policy Alternatives. *Landlessness and Land Rights in Post-Tsunami Sri Lanka*. https://www.cpalanka.org/wp-content/uploads/2007/08/IFRC_land_study.pdf (2005).

29. Huang, S.-M. Heritage and Postdisaster Recovery: Indigenous Community Resilience. *Nat. Hazards Rev.* 19, 05018008 (2018).

30. Kondo, T. Miyagi Prefecture, Sendai, Japan. in *Landscape Architecture for Sea Level Rise: Innovative Global Solutions* 154–164 (2022). doi:10.4324/9781003183419-19.

31. Pinter, N. *et al.* Large-scale managed retreat and structural protection following the 2011 Japan tsunami. *J. Int. Soc. Prev. Mitig. Nat. Hazards* 96, 1429–1436 (2019).

32. Tiong-Sa, T. & Bird, C. F. Managed retreat of a mangrove coast: A case study at Sungai Lurus, Peninsular Malaysia. *Asian Geogr.* 14, 71–85 (1995).

33. Vinh, T. T., Kant, G., Huan, N. N. & Pruszak, Z. Sea dike erosion and coastal retreat at Nam Ha Province, Vietnam. in vol. 3 2820–2828 (1997).

34. Prasain, S. Climate change adaptation measure on agricultural communities of Dhye in Upper Mustang, Nepal. *Climatic change* 148, 279–291 (2018).

35. Correa, E. *Preventive Resettlement of Populations at Risk of Disaster Experiences from Latin America*. (The World Bank, Washington DC, 2011).

36. Yarina, L., Mazereeuw, M. & Ovalles, L. A retreat critique: Deliberations on design and ethics in the flood zone. *J. Landsc. Archit.* 14, 8–23 (2019).

37. Rodriguez Del Valle, L. ENLACE Caño Martín Peña: A Restoration and Resiliency Project - An opportunity to transform the San Juan Metropolitan Area. (2017).

38. ENLACE Landscape Architecture. *Marvel* https://marveldesigns.com/work/enlace-landscape-architecture/340.

39. Corporación del Proyecto ENLACE. *Transforming a City*. https://cano3punto7.org/pdf/transforming.pdf (2014).

40. Gini, G., Cardoso, T. M. & Ramos, E. P. When the two seas met: preventive and self-managed relocation of the Nova Enseada community in Brazil. *Forced Migration Review* 64, 35–38 (2020).

41. Millington, N. Stormwater Politics: Flooding, Infrastructure, and Urban Political Ecology in São Paulo, Brazil. *Water Altern.* 14, (2021).

42. Portz, L., Manzolli, R. P. & Alcántara-Carrió, J. Dune system restoration in Osório municipality (Rio grande do sul, Brazil): Good practices based on coastal management legislation. in *Coastal Research Library* vol. 24 41–58 (2018).

43. Correa, I. D. & Gonzalez, J. L. Coastal erosion and village relocation: a Colombian case study. *Ocean Coast. Manag.* 43, 51–64 (2000).

44. Williams, A. T., Rangel-Buitrago, N., Pranzini, E. & Anfuso, G. The management of coastal erosion. *Ocean Coast. Manag.* 156, 4–20 (2018).

45. Bergmann, J. Planned relocation in Peru: advancing from well-meant legislation to good practice. *J. Environ. Stud. Sci.* 11, 365–375 (2021).

46. Nelson, M. *et al. Beyond Buyouts: Adaptive Migration and the Need for Equitable Relocation Strategies*. https://doi.org/10.13140/RG.2.2.22051.37925 (2020).

47. Goldenberg, S. Alaska on the edge: Newtok's residents race to stop village falling into sea. *the Guardian* (2013).

48. LeMay, P. *Newtok Planning Group Meeting April 25, 2022 Meeting Notes*. https://www.commerce.alaska.gov/web/Portals/4/pub/Newtok%20Planning%20Group/2022.0425_NPG_Meeting_Notes_Final.pdf (2022).

49. Cottar, S., Doberstein, B., Henstra, D. & Wandel, J. Evaluating property buyouts and disaster recovery assistance (Rebuild) options in Canada: A comparative analysis of Constance Bay, Ontario and Pointe Gatineau, Quebec. *Nat. Hazards* 109, 201–220 (2021).

50. Carey, J. Core Concept: Managed retreat increasingly seen as necessary in response to climate change's fury. *Proc. Natl. Acad. Sci. U. S. A.* 117, 13182–13185 (2020).

51. Park People. Master Plan for the Development of Pointe-Gatineau and Lac-Beauchamp Vacant Lands. *Park People Canada* https://parkpeople.ca/resources/case-study/"https://parkpeople.ca/resources/case-study/master-plan-for-the-development-of-pointe-gatineau-and-lac-beauchamp-vacant-lands".

52. Saunders-Hastings, Barnard, & Doberstein. *Planned Retreat Approaches to Support Resilience to Climate Change in Canada*. https://geoscan.nrcan.gc.ca/starweb/geoscan/servlet.starweb?path=geoscan/fulle.web&search1=R=328323 (2020) doi:10.4095/328323.

53. High River Council. Urban Resilience, Innovation and Adaptation Ponds, Parks and People. https://static1.squarespace.com/static/5977ae4ef14aa1a84a5f2bad/t/5bb3cc7f15fcc0b3bd296206/1538509955949/Tour+Agenda+info+package.pdf.

54. Kovacs, P., Guilbault, S., Darwish, L. & Comella, M. *Cities Adapt to Extreme Weather: Celebrating Local Leadership*. https://www.iclr.org/wp-content/uploads/2018/12/cities-adapt-tp-extreme-weather-web-moncton-edit.pdf.

55. Virgin, S. D. S. *et al.* A managed realignment in the upper Bay of Fundy: Community dynamics during salt marsh restoration over 8 years in a megatidal, ice-influenced environment. *Ecol. Eng.* 149, (2020).

56. Maximizing the Benefits of Managed Realignment in Truro. *CBWES Inc.* https://www.cbwes.com/work/project-frm-truro.

57. Onslow-North River Managed Realignment and Tidal Wetland Restoration. *TransCoastal Adaptations* https://www.transcoastaladaptations.com/onslow-north-river.

58. OECD. *Responding to Rising Seas: OECD Country Approaches to Tackling Coastal Risks*. (OECD Publishing, Paris, France, 2019).

59. Zavar, E. An analysis of floodplain buyout memorials: four examples from central U.S. floods of 1993–1998. *GeoJournal* 84, 135–146 (2019).

60. FEMA. Valmeyer Acquisitions Buyouts Create Open Space | FEMA.gov. https://www.fema.gov/case-study/valmeyer-acquisitions-buyouts-create-open-space (2021).

61. Elam, S. A flood forced this town to move. It could be a model for others hit by the climate crisis. *CNN* https://www.cnn.com/2019/07/17/us/valmeyer-flooding-climate-crisis-midwest/index.html (2019).

62. Koslov, L. The case for retreat. *Public Cult.* 28, 359–387 (2016).

63. Tate, E., Strong, A., Kraus, T. & Xiong, H. Flood recovery and property acquisition in Cedar Rapids, Iowa. *Nat. Hazards* 80, 2055–2079 (2016).

64. Siders, A. R. Social justice implications of US managed retreat buyout programs. *Clim. Change* 152, 239–257 (2019).

65. Spidalieri, K. & Smith, I. *Managing the Retreat from Rising Seas City of Austin, Texas: Flood Risk Reduction Buyout Projects*. https://www.georgetownclimate.org/files/MRT/GCC_20_Austin-5web.pdf (2020).

66. NYC Department of Housing Preservation and Development. *Resilient Edgemere Community Plan*. https://www.nyc.gov/assets/hpd/downloads/pdfs/services/resilient-edgemere-report.pdf (2017).

67. New York City. Oct 2017 Build it Back Progress Report. https://www.nyc.gov/assets/housingrecovery/downloads/pdf/2017/october_2017_build_it_back_progress_update.pdf.

68. Seip, M. & Borrero, D. *Community Visioning for Vacant Land Following Managed Retreat in Edgemere, Queens, N.Y.* https://www.riserockaway.org/rise/initiatives/community-visioning-for-vacant-l/community-visioning-edgemere/CVE-final-report-action-plan:en-us.pdf.

69. Floodplains by Design. Floodplains by Design. https://floodplainsbydesign.org/ (2023).

70. Floodplains by Design. Impact | Floodplains by Design. https://floodplainsbydesign.org/communities-in-action/impact/.

71. Floodplains by Design. *Ranked List and Program Highlights*. https://walandtrusts.org/wordpress/wp-content/uploads/2023/01/FbD-Ranked-List-and-Program-Highlights.pdf.

72. Shi, L. *et al.* Equitable buyouts? Learning from state, county, and local floodplain management programs. *Clim. Change* 174, (2022).

73. Greer, A. & Brokopp Binder, S. A historical assessment of home buyout policy: Are we learning or just failing? *Hous. Policy Debate* 27, 372–392 (2017).

74. Becker, W. *Come Rain, Come Shine: A Case Study of a Floodplain Relocation Project at Soldiers Grove, Wisconsin.* (Bureau of Water Regulation and Zoning, Wisconsin Department of Natural Resources, Wisconsin, 1983).

75. David, E. & Mayer, J. Comparing Costs of Alternative Flood Hazard Mitigation Plans The Case of Soldiers Grove, Wisconsin. *J. Am. Plann. Assoc.* 50, 22–35 (1984).

76. FEMA. Small Wisconsin Villages Leads the Nation: Rebuilds Above Floodwaters | FEMA.gov. https://www.fema.gov/case-study/small-wisconsin-villages-leads-nation-rebuilds-above-floodwaters.

77. Adaptation Clearinghouse. Caltrans Devil's Slide Realignment Project. https://www.adaptationclearinghouse.org/resources/caltrans-devil-eys-slide-realignment-project.html.

78. Spidalieri, K., Smith, I., Grannis, J., Li, J. & Lov, A. Managing the Retreat from Rising Seas.

79. City of Portland. Foster Floodplain Natural Area | Projects and Programs | The City of Portland, Oregon. https://www.portlandoregon.gov/bes/article/286175.

80. City of Punta Gorda. City of Punta Gorda Adaptation Plan Update.

81. Mecklenburg County government. Floodplain Buyout Program | Storm Water Services. https://stormwaterservices.mecknc.gov/floodplain-buyout-program.

82. City of Greenville. *City of Greenville: Flood Land Reuse Plan*. https://www.greenvillenc.gov/Home/ShowDocument?id=6674 (2004).

83. City of Kinston. *Retrofitting Green Conservation Plan*. https://www.ci.kinston.nc.us/DocumentCenter/View/4647/Retrofitting-Green-Conservation-Plan-2005 (2005).

84. FEMA. *FEMA Mitigation Case Studies, Innovative Floodplain Management - Kinston*. https://www.fema.gov/pdf/casestudys/kinston_cs.pdf.

85. Hurricane Matthew Disaster Recovery and Resilience Initiative, University of. *Land Suitability Analysis for Post-Disaster Housing Relocation - Briefing for the City of Kinston, NC*. (2018).

86. U.S. Department of Housing and Urban Development. Preparing for the Next Disaster: Three Models of Building Resilient Communities | HUD USER. https://www.huduser.gov/portal/periodicals/em/winter15/highlight3.html (2015).

87. U.S Environmental Protection Agency. *Smart Growth Fixes for Climate Adaptation and Resilience*. https://www.epa.gov/sites/default/files/2017-01/documents/smart_growth_fixes_climate_adaptation_resilience.pdf (2017).

88. City of Grand Forks. *The Greenway 2012 Supplement*. (2012).

89. Schwartz, J. Surrendering to Rising Seas. *Scientific American* https://www.scientificamerican.com/article/surrendering-to-rising-seas/ (2018).

90. University of North Carolina Institute for the Environment. *Sayreville Case Study*. https://www.eli.org/sites/default/files/eli-pubs/sayrevillecasestudy.pdf (2016).

91. Freudenburg, R., Calvin, E., Tolkoff, L. & Brawley, D. *Buy-in for Buyouts the Case for Managed Retreat from Flood Zones*. (2016).

92. Spidalieri, K. *Greauxing Resilience at Home: City of Gonzales, Louisiana*. (2022).

93. Nelson, M. Using Land Swaps to Concentrate Redevelopment and Expand Resettlement Options in Post-Hurricane Katrina New Orleans. *J. Am. Plann. Assoc.* 80, 426–437 (2014).

94. Adaptation Clearinghouse. New Orleans, Louisiana Project Home Again Land Swaps | Adaptation Clearinghouse. https://www.adaptationclearinghouse.org/resources/new-orleans-louisiana-project-home-again-land-swaps.html.

95. Marshall, J. T. Weathering NEPA Review: Superstorms and Super Slow Urban Recovery. *Ecol. Law Q.* 41, 81–130 (2014).

96. Cohn, J. L. *et al.* Strategies to work towards long-term sustainability and resiliency of nature-based solutions in coastal environments: A review and case studies. *Integr. Environ. Assess. Manag.* 18, 123–134 (2022).

97. University of North Carolina Institute for the Environment. *Wayne Case Study*. https://www.eli.org/sites/default/files/eli-pubs/waynecasestudy.pdf (2016).

98. Binder, S. B. *et al.* Limbo: the unintended consequences of home buyout programmes on peripheral communities. *Environ. Hazards* 19, 488–507 (2020).

99. Pinter, N. & Rees, J. C. Assessing managed flood retreat and community relocation in the Midwest USA. *Nat. Hazards* 107, 497–518 (2021).

100. *Pioneer Bank - Media Collections Online, Old Shawneetown, Indiana University Audio-Visual Center*.

101. Haas, E. 30 years later: How Pattonsburg survived the 1993 flood by moving the town uphill. *Columbia Missourian* https://www.columbiamissourian.com/news/state_news/30-years-later-how-pattonsburg-survived-the-1993-flood-by-moving-the-town-uphill/article_4c07fe14-ed0d-11ed-bbf0-8be6b15d4878.html (2023).

102. Village Relocates Uphill Above Floodplain | FEMA.gov. https://www.fema.gov/case-study/village-relocates-uphill-above-floodplain (2021).

103. Kerlin, K. Small Towns, Big Flood Waters. *UC Davis* https://www.ucdavis.edu/climate/news/small-towns-big-flood-waters (2019).

104. Bragg, W. K., Gonzalez, S. T., Rabearisoa, A. & Stoltz, A. D. Communicating managed retreat in California. *Water Switz.* 13, (2021).

105. Kochnower, D., Reddy, S. M. W. & Flick, R. E. Factors influencing local decisions to use habitats to protect coastal communities from hazards. *Ocean Coast. Manag.* 116, 277–290 (2015).

106. Griggs, G. & Patsch, K. The Protection/Hardening of California's Coast: Times Are Changing. *J. Coast. Res.* 35, 1051–1061 (2019).

107. Grace-McCaskey, C. A., Pearce, S. C., Harris, L., Corra, M. & Evans, K. J. Finding voices in the floods of Freedom Hill: innovating solutions in Princeville, North Carolina. *J. Environ. Stud. Sci.* 11, 341–351 (2021).

108. Nelson, K. S. & Camp, J. Quantifying the Benefits of Home Buyouts for Mitigating Flood Damages. *Anthropocene* 31, (2020).

109. Conrad, D., McNitt, B. & Stout, M. *A Report on Voluntary Property Buyouts in the Nation's Floodplains, A Common Ground Solution Serving People at Risk, Taxpayers and the Environment.* (1998).

110. Kodis, M., Bortman, M. & Newkirk, S. Strategic retreat for resilient and equitable climate adaptation: the roles for conservation organizations. *J. Environ. Stud. Sci.* 11, 493–502 (2021).

111. Haley, M. X. & Siegel, J. D. The Squibnocket Causeway and Beach Restoration Projects: Managed Retreat and Restoration of Coastal Beach. in vols 2021-November 339–348 (2021).

112. Loughran, K. & Elliott, J. R. Unequal Retreats: How Racial Segregation Shapes Climate Adaptation. *Hous. Policy Debate* 32, 171–189 (2022).

113. Harris County. HCFCD Voluntary Buyout Guidance. https://www.hcfcd.org/Portals/62/Home-Buyout-Program/FINAL%20HCFCD%20Voluntary%20Buyout%20Guidance_20200305a.pdf?ver=2020-03-11-201520-707 (2020).

114. Kousky, C. Managing shoreline retreat: A US perspective. *Clim. Change* 124, 9–20 (2014).

115. Seebauer, S. & Winkler, C. Coping strategies and trajectories of life satisfaction among households in a voluntary planned program of relocation from a flood-risk area. *Clim. Change* 162, 2219–2239 (2020).

116. Thaler, T. & Fuchs, S. Financial recovery schemes in Austria: how planned relocation is used as an answer to future flood events. *Environ. Hazards* 19, 268–284 (2020).

117. Thaler, T., Seebauer, S. & Schindelegger, A. Patience, persistence and pre-signals: Policy dynamics of planned relocation in Austria. *Glob. Environ. Change* 63, (2020).

118. Thaler, T. Just retreat—how different countries deal with it: examples from Austria and England. *J. Environ. Stud. Sci.* 11, 412–419 (2021).

119. Walsh, C., Lennon, M., Scott, M. & Tubridy, F. Spatial imaginaries in flood risk management: insights from a managed retreat initiative in upper Bavaria. *J. Environ. Plan. Manag.* (2022) doi:10.1080/09640568.2022.2082927.

120. Mayr, B., Thaler, T. & Hübl, J. Successful Small-Scale Household Relocation after a Millennial Flood Event in Simbach, Germany 2016. *Water* 12, 156 (2020).

121. Eva Weisner & Gerald Schernewski. Adaptation to climate change: A combined coastal protection and re-alignment scheme in a Baltic tourism region. *J. Coast. Res.* 65, 1963–1968 (2013).

122. Schernewski, G., Bartel, C., Kobarg, N. & Karnauskaite, D. Retrospective assessment of a managed coastal realignment and lagoon restoration measure: the Gelringer Birk, Germany. *J. Coast. Conserv.* 22, 157–167 (2018).

123. Hofstede, J. L. A. On the feasibility of managed retreat in the Wadden Sea of Schleswig-Holstein. *J. Coast. Conserv.* 23, 1069–1079 (2019).

124. van den Hoven, K., Kroeze, C. & van Loon-Steensma, J. M. Characteristics of realigned dikes in coastal Europe: Overview and opportunities for nature-based flood protection. *Ocean Coast. Manag.* 222, (2022).

125. Rupp-Armstrong, S. Coastal and estuarine retreat: A comparison of the application of managed realignment in England and Germany. *J. Coast. Res.* 23, 1418–1430 (2007).

126. Esteves, L. S. & Williams, J. J. Managed Realignment in Europe: A Synthesis of Methods, Achievements, and Challenges. in *Living Shorelines: The Science and Management of Nature-Based Coastal Protection* 157–182 (2017). doi:10.1201/9781315151465-11.

127. Schut, M., Leeuwis, C. & van Paassen, A. Room for the River: Room for Research? The case of depoldering De Noordwaard, the Netherlands. *Sci. Public Policy* 37, 611–627 (2010).

128. Lisa Danielson & Michael Mullan. The challenge of coastal adaptation. in *Responding to Rising Seas OECD Country Approaches to Tackling Coastal Risks* (2019).

129. Sousa, C. A. M., Cunha, M. E. & Ribeiro, L. Tracking 130 years of coastal wetland reclamation in Ria Formosa, Portugal: Opportunities for conservation and aquaculture. *Land Use Policy* 94, 104544 (2020).

130. Gee, N. Managing the Anglian coast. *Geogr. Rev.* 21, 2–5 (2008).

131. Strack, M. The order is rapidly fadin': Responding to the impact of climate change on property with reference to the Aotearoa New Zealand context. *J. Prop. Plan. Environ. Law* 12, 19–34 (2019).

132. DEFRA. *Coastal Pathfinder Evaluation: An Assessment of the Five Largest Pathfinder Projects*. (2011).

133. Hazelden, J. & Boorman, L. A. Soils and 'managed retreat' in South East England. *Soil Use Manag.* 17, 150–154 (2001).

134. Townend, I. & Pethick, J. Estuarine flooding and managed retreat. *Philos. Trans. R. Soc. Math. Phys. Eng. Sci.* 360, 1477–1495 (2002).

135. Rupp-Armstrong, S. & Nicholls, R. J. Coastal and estuarine retreat: A comparison of the application of managed realignment in England and Germany. *J. Coast. Res.* 23, 1418–1430 (2007).

136. Emmerson, R. H. C., Birkett, J. W., Scrimshaw, M. & Lester, J. N. Solid phase partitioning of metals in managed retreat soils: Field changes over the first year of tidal inundation. *Sci. Total Environ.* 254, 75–92 (2000).

137. Emmerson, R. H. C., Manatunge, J. M. A., Macleod, C. L. & Lester, J. N. Tidal Exchanges Between Orplands Managed Retreat Site and the Blackwater Estuary, Essex. *Water Environ. J.* 11, 363–372 (1997).

138. May, A. & Smart, D. Managed retreat of the Essex coast. *Geogr. Rev.* 17, 38–41 (2003).

139. NCCARF. Managed coastal realignment projects in the UK. Snapshot for CoastAdapt.,. *National Climate Change Adaptation Research Facility, Gold Coast.* https://coastadapt.com.au/sites/default/files/case_studies/SS46_UK_Coastal_Realignment.pdf (2017).

140. Abbotts Hall | Essex Wildlife Trust. https://www.essexwt.org.uk/nature-reserves/abbotts-hall.

141. National Trust. Plan your visit to Northey Island | Essex. *National Trust* https://www.nationaltrust.org.uk/visit/essex-bedfordshire-hertfordshire/northey-island/plan-your-visit-to-northey-island.

142. Brown, I. Do habitat compensation schemes to offset losses from sea level rise and coastal squeeze represent a robust climate change adaptation response? *Ocean Coast. Manag.* 219, (2022).

143. McAlinden, B. Managed realignment at Steart, Somerset. *Institution of Civil Engineers (ICE)* https://www.ice.org.uk/engineering-resources/case-studies/managed-realignment-at-steart-somerset/ (2023).

144. Mossman, H. L. *et al.* Rapid carbon accumulation at a saltmarsh restored by managed realignment exceeded carbon emitted in direct site construction. *PLOS ONE* 17, e0259033 (2022).

145. Steart Marshes Rewilding Project. *Rewilding Britain* https://www.rewildingbritain.org.uk/rewilding-projects/steart-marshes.

146. Beefing up protection of the Bleadon Levels | Wessex Water. *Wessex Water Services Ltd* https://www.wessexwater.co.uk/news/beefing-up-protection-of-the-bleadon-levels.

147. Myatt-Bell, L. B., Scrimshaw, M. D., Lester, J. N. & Potts, J. S. Public perception of managed realignment: Brancaster West Marsh, North Norfolk, UK. *Mar. Policy* 26, 45–57 (2002).

148. Royal Society for the Protection of Birds. Freiston Shore. https://www.rspb.org.uk/days-out/reserves/freiston-shore (2023).

149. Myatt, L. B., Scrimshaw, M. D. & Lester, J. N. Public perceptions and attitudes towards a forthcoming managed realignment scheme: Freiston Shore, Lincolnshire, UK. *Ocean Coast. Manag.* 46, 565–582 (2003).

150. ABP Marine Environmental Research Ltd. *Wallasea Island North Bank Realignment: Environmental Statement*. (2004).

151. Wallasea Island Nature Reserve, Essex. https://www.rspb.org.uk/days-out/reserves/wallasea-island.

152. ABPmer. Wallasea case studies OMREG database. https://www.omreg.net/query-database/?fromnav=1&q=wallasea.

153. ABPmer. Cone Pill. https://www.omreg.net/query-database/88-cone-pill/.

154. Andrews, J. E. *et al.* Biogeochemical value of managed realignment, Humber estuary, UK. *Sci. Total Environ.* 371, 19–30 (2006).

155. Yorkshire Wildlife Trust. Paull Holme Strays Nature Reserve. https://www.ywt.org.uk/nature-reserves/paull-holme-strays-nature-reserve.

156. Royal Society for the Protection of Birds. Titchwell Marsh. https://www.rspb.org.uk/days-out/reserves/titchwell-marsh.

157. Royal Society for the Protection of Birds. Ynys-hir. https://www.rspb.org.uk/days-out/reserves/ynys-hir.

158. MacDonald, M. A., de Ruyck, C., Field, R. H., Bedford, A. & Bradbury, R. B. Benefits of coastal managed realignment for society: Evidence from ecosystem service assessments in two UK regions. *Estuar. Coast. Shelf Sci.* 244, (2020).

159. Royal Society for the Protection of Birds. Hesketh Out Marsh Facilities. https://www.rspb.org.uk/days-out/reserves/hesketh-out-marsh/facilities.

160. UK Government. Flood risk reduced and wildlife brimming over on the Ribble. https://www.gov.uk/government/news/flood-risk-reduced-and-wildlife-brimming-over-on-the-ribble.

161. McAlinden, B. Managed realignment at Medmerry, Sussex. *Institution of Civil Engineers (ICE)* https://www.ice.org.uk/engineering-resources/case-studies/managed-realignment-at-medmerry-sussex/ (2023).

162. Royal Society for the Protection of Birds. Medmerry facilities. https://www.rspb.org.uk/days-out/reserves/medmerry/facilities.

163. Bone, D. A. Selsey and Bracklesham, West Sussex: A case study of the impact of coastal management on foreshore exposures and geological conservation. *Proc. Geol. Assoc.* 131, 432–442 (2020).

164. Rocle, N., Dachary-Bernard, J. & Rey-Valette, H. Moving towards multi-level governance of coastal managed retreat: Insights and prospects from France. *Ocean Coast. Manag.* 213, (2021).

165. Fouqueray, T., Trommetter, M. & Frascaria-Lacoste, N. Managed retreat of settlements and infrastructures: ecological restoration as an opportunity to overcome maladaptive coastal development in France. *Restor. Ecol.* 26, 806–812 (2018).

166. Albert, S. *et al.* Heading for the hills: climate-driven community relocations in the Solomon Islands and Alaska provide insight for a 1.5 °C future. *Reg. Environ. Change* 18, 2261–2272 (2018).

167. *Vunidogoloa Moves Higher – Climate Change Relocation*. (2020).

168. Tronquet, C. *The State of Environmental Migration 2015: From Vunidogoloa to Kenani: An Insight into Successful Relocation*. (2015).

169. Yee, M., Piggott-Mckellar, A. E., McMichael, C. & McNamara, K. E. Climate Change, Voluntary Immobility, and Place-Belongingness: Insights from Togoru, Fiji. *Climate* 10, (2022).

170. McMichael, C. & Powell, T. Planned Relocation and Health: A Case Study from Fiji. *Int. J. Environ. Res. Public. Health* 18, 4355 (2021).

171. Piggott-McKellar, A. E. & McMichael, C. The immobility-relocation continuum: Diverse responses to coastal change in a small island state. *Environ. Sci. Policy* 125, 105–115 (2021).

172. Bertana, A. Relocation as an Adaptation to Sea-Level Rise: Valuable Lessons from the Narikoso Village Relocation Project in Fiji. *Case Stud. Environ.* 3, 1–7 (2019).

173. Ruggieri, B. Moving to higher ground: Planning for relocation as an adaptation strategy to climate change in the Fiji Islands. in *Global Views on Climate Relocation and Social Justice* (Routledge, 2021).

174. Martin, P. C. M., Nunn, P., Leon, J. & Tindale, N. Responding to multiple climate-linked stressors in a remote island context: The example of Yadua Island, Fiji. *Clim. Risk Manag.* 21, 7–15 (2018).

175. Okada, T., Haynes, K., Bird, D., van den Honert, R. & King, D. Recovery and resettlement following the 2011 flash flooding in the Lockyer Valley. *Int. J. Disaster Risk Reduct.* 8, 20–31 (2014).

176. Queensland Reconstruction Authority. Rebuilding Grantham together: Development Scheme Grantham Reconstruction Area. https://www.qra.qld.gov.au/sites/default/files/2018-10/rebuilding-grantham-full.pdf (2011).

177. Vandenbeld, A. Fostering community acceptance of managed retreat in New Zealand. in *Climate Adaptation Futures* 161–166 (Wiley-Blackwell, Somerset, GB, 2013).

178. Hanna, C. Restraints of change: Limits to 'managed retreats' in Aotearoa New Zealand. (The University of Waikato, Hamilton, New Zealand, 2019).

179. Hanna, C., Cretney, R. & White, I. Re-Imagining Relationships with Space, Place, and Property: The Story of Mainstreaming Managed Retreats in Aotearoa-New Zealand. *Plan. Theory Pract.* 23, 681–702 (2022).

180. Project Twin Streams. Project Twin Streams Lucinda Place Community Orchard. https://projecttwinstreams.com/where/glen-eden/lucinda-place-community-orchard/.

181. Project Twin Streams. Project Twin Streams Pā Harakeke. https://projecttwinstreams.com/where/opanuku-stream/pa-harekeke-_newabc/.

182. Project Twin Streams. Lower Opanuku trail. https://projecttwinstreams.com/wp-content/uploads/2012/10/Lower-Opanuku.pdf.

183. Atlas Communications & Media Ltd. *Project Twin Streams Case Study: Largescale Property Purchase without Recourse to Compulsory Purchase*. (2011).

184. Waikato District Council. Port Waikato erosion. (2020).

185. Waikato District Council. Port Waikato Update 5 September 2019. https://www.waikatodistrict.govt.nz/docs/default-source/your-distric/port-waikato-erosion/port-waikato-update---5-september-2019.pdf?sfvrsn=bb468b22.

186. Ebenoh, J. *Sustainability and Wellbeing Committee - Adaptive Management Planning and Port Waikato Erosion*. https://www.waikatodistrict.govt.nz/docs/default-source/your-distric/port-waikato-erosion/port-waikato/adaptive-management-planning-and-port-waikato-erosion-report-june-2023.pdf?sfvrsn=e7cc69c8_1.

187. Water New Zealand. *Water Infrastructure Focus - Re-Building a Community*. https://www.waternz.org.nz/Attachment?Action=Download&Attachment_id=4265.

188. Corlett, E. Edgecumbe retirees near stop bank face new mortgage. *RNZ* https://www.rnz.co.nz/news/national/333212/edgecumbe-retirees-near-stop-bank-face-new-mortgage (2017).

189. Desmarais, F. Managed retreat for Kāpiti Coast park will go ahead. https://www.stuff.co.nz/environment/climate-news/116270157/managed-retreat-for-kpiti-coast-park-will-go-ahead.

190. One News. Paekākāriki Surf Club demolished after 60 years due to erosion, climate. *1 News* https://www.1news.co.nz/2022/11/25/paekakariki-surf-club-demolished-after-60-years-due-to-erosion-climate/.

191. Christchurch City Council. Residential Red Zone. *Christchurch City Council* https://ccc.govt.nz/parks-and-gardens/explore-parks/residential-red-zone (2023).

192. Harvie, W. Some Port Hills red zone land may be sold off if public uses not found. *Stuff* https://www.stuff.co.nz/the-press/news/300387349/some-port-hills-red-zone-land-may-be-sold-off-if-public-uses-not-found (2021).

193. Law, T. Council considers selling Port Hills red zone land once deemed too dangerous to build on. *Stuff* https://www.stuff.co.nz/the-press/news/131611738/council-considers-selling-port-hills-red-zone-land-once-deemed-too-dangerous-to-build-on (2023).

194. Waimakariri District Council. Waimakariri Residential Red Zone Recovery Plan - He Mahere Whakarauora i te Whenua Rāhui o Waimakariri. https://www.waimakariri.govt.nz/__data/assets/pdf_file/0025/139354/DPMCWRRZRecoveryPlanWeb.pdf (2016).

195. Matthewman, S. & Goode, L. City of quakes: Excavating the future in christchurch. *N. Z. Sociol.* 35, 77–98 (2020).

196. Christchurch City Council. *Christchurch District Plan*. https://districtplan.ccc.govt.nz/pages/plan/Book.aspx?exhibit=DistrictPlan&hid=87913 (2017).

197. Regenerate Christchurch. *Ōtākaro Avon River Corridor Regeneration Plan*. https://www.dpmc.govt.nz/sites/default/files/2019-08/Otakaro%20Avon%20River%20Corridor%20Regeneration%20PlanReducedSize.pdf (2019).

198. Noy, I. Paying a price of climate change: Who pays for managed retreats? *Curr. Clim. Change Rep.* 6, 17–23 (2020).

199. Saunders, W. S. A. & Becker, J. S. A discussion of resilience and sustainability: Land use planning recovery from the Canterbury earthquake sequence, New Zealand. *Int. J. Disaster Risk Reduct.* 14, 73–81 (2015).

200. Law, T. Christchurch City Council gets ready to demolish handful of Flockton properties | Stuff.co.nz. https://www.stuff.co.nz/the-press/news/99498717/christchurch-city-council-gets-ready-to-demolish-handful-of-flockton-properties (2017).

201. Hanna, C., White, I. & Glavovic, B. The uncertainty contagion: Revealing the interrelated, cascading uncertainties of managed retreat. *Sustain. Switz.* 12, (2020).

202. Whakatāne District Council. Chapter 3 - Operative District Plan - 17 December 2021. https://www.whakatane.govt.nz/sites/www.whakatane.govt.nz/files/documents/documents-section/council-plans/operative-district-plan/2017-chapters/Chapter%203%20-%20Operative%20District%20Plan%20-%2017%20December%202021%20-%20NPS-UD%20Mininum%20parking%20rules%20removal.pdf (2021).

203. Stroombergen, A. & Lawrence, J. A novel illustration of real options analysis to address the problem of probabilities under deep uncertainty and changing climate risk. *Clim. Risk Manag.* 38, (2022).

204. Campbell, A., Allan, A., Dobson, B. & Kessing, V. Building resilience into a rigid flood management system: Lessons from the Riverlink project. (2017).

205. Hutt City Council. *Central City Transformation Plan*.
https://hccpublicdocs.azurewebsites.net/api/download/587890ba7b2542ef8532867346c3a343/_extcomms/77094e8820840be24831bd087183bf6449b9 (2019).

206. Hayward, B. 'Nowhere far from the sea': Political challenges of coastal adaptation to climate change in New Zealand. *Polit. Sci.* 60, 47–59 (2008).

207. Edwards, J. B. The logistics of climate-induced resettlement: Lessons from the Carteret Islands, Papua New Guinea. *Refug. Surv. Q.* 32, 52–78 (2013).

208. UNDP. *Tulele Peisa Papua New Guinea: Equator Initiative Case Study Series*. (2016).
